# Genotype–phenotype correlations within the *Geodermatophilaceae*

**DOI:** 10.3389/fmicb.2022.975365

**Published:** 2022-11-10

**Authors:** Maria del Carmen Montero-Calasanz, Adnan Yaramis, Manfred Rohde, Peter Schumann, Hans-Peter Klenk, Jan P. Meier-Kolthoff

**Affiliations:** ^1^IFAPA Las Torres-Andalusian Institute of Agricultural and Fisheries Research and Training, Junta de Andalucía, Seville, Spain; ^2^School of Natural and Environmental Sciences, Newcastle University, Newcastle upon Tyne, United Kingdom; ^3^Central Facility for Microscopy, HZI – Helmholtz Centre for Infection Research, Braunschweig, Germany; ^4^Leibniz Institute DSMZ – German Collection of Microorganisms and Cell Cultures, Braunschweig, Germany; ^5^Department Bioinformatics and Databases, Leibniz Institute DSMZ – German Collection of Microorganisms and Cell Cultures, Braunschweig, Germany

**Keywords:** *Trujillonella*, *Pleomorpha*, *Goekera*, phylogenetic systematics, *in silico* chemotaxonomy

## Abstract

The integration of genomic information into microbial systematics along with physiological and chemotaxonomic parameters provides for a reliable classification of prokaryotes. *In silico* analysis of chemotaxonomic traits is now being introduced to replace characteristics traditionally determined in the laboratory with the dual goal of both increasing the speed of the description of taxa and the accuracy and consistency of taxonomic reports. Genomics has already successfully been applied in the taxonomic rearrangement of *Geodermatophilaceae (Actinomycetota)* but in the light of new genomic data the taxonomy of the family needs to be revisited. In conjunction with the taxonomic characterisation of four strains phylogenetically located within the family, we conducted a phylogenetic analysis of the whole proteomes of the sequenced type strains and established genotype–phenotype correlations for traits related to chemotaxonomy, cell morphology and metabolism. Results indicated that the four isolates under study represent four novel species within the genus *Blastococcus*. Additionally, the genera *Blastococcus*, *Geodermatophilus* and *Modestobacter* were shown to be paraphyletic. Consequently, the new genera *Trujillonella*, *Pleomorpha* and *Goekera* were proposed within the *Geodermatophilaceae* and *Blastococcus endophyticus* was reclassified as *Trujillonella endophytica* comb. nov., *Geodermatophilus daqingensis* as *Pleomorpha daqingensis* comb. nov. and *Modestobacter deserti* as *Goekera deserti* comb. nov. Accordingly, we also proposed emended descriptions of *Blastococcus aggregatus, Blastococcus jejuensis, Blastococcus saxobsidens* and *Blastococcus xanthilyniticus*. *In silico* chemotaxonomic results were overall consistent with wet-lab results. Even though *in silico* discriminatory levels varied depending on the respective chemotaxonomic trait, this approach is promising for effectively replacing and/or complementing chemotaxonomic analyses at taxonomic ranks above the species level. Finally, interesting but previously overlooked insights regarding morphology and ecology were revealed by the presence of a repertoire of genes related to flagellum synthesis, chemotaxis, spore production and pilus assembly in all representatives of the family. A rich carbon metabolism including four different CO_2_ fixation pathways and a battery of enzymes able to degrade complex carbohydrates were also identified in *Blastococcus* genomes.

## Introduction

The family *Geodermatophilaceae,* belonging to order *Geodermatophilales* (Sen et al., 2014), was initially proposed by Normand et al. (1996), confirmed by Stackebrandt et al. (1997), formally described by Normand (2006) and later emended by Zhi et al. (2009) and Montero-Calasanz et al. (2017). The family accommodates the genera *Blastococcus* (Ahrens and Moll, 1970; Urzì et al., 2004; Lee, 2006; Hezbri et al., 2016), *Geodermatophilus*, the type genus (Luedemann, 1968; Montero-Calasanz et al., 2017; Montero-Calasanz, 2020a), *Klenkia* (Montero-Calasanz et al., 2017), and *Modestobacter* (Mevs et al., 2000; Reddy et al., 2007; Xiao et al., 2011; Qin et al., 2013; Montero-Calasanz et al., 2019).

The genus *Blastococcus* consists of Gram-reaction-positive aerobic bacteria that can be distinguished by the presence of individual motile rod-shaped cells and/or non-motile cocci (Lee, 2006) with a tendency to form aggregates (Urzì et al., 2004; Hezbri et al., 2016). At the time of writing, the genus comprises 10 validly named species including *B. aggregatus* (type species), isolated from sediment from Baltic Sea (Ahrens and Moll, 1970), *B. atacamensis*, isolated from an extreme hyper-arid Atacama Desert soil (Castro et al., 2018), *B. capsensis,* isolated from an ancient Roman pool in Tunisia (Hezbri et al., 2016), *B. colisei,* isolated from the ruins of a Roman amphitheatre in Tunisia (Hezbri et al., 2017), *B. deserti,* isolated from Gurbantunggut Desert in China (Yang et al., 2019), *B. endophyticus,* isolated from Chinese medicinal plant leaves (Zhu et al., 2013; Hezbri et al., 2016), *B. jejuensis,* isolated from sand in Korea (Lee, 2006; Hezbri et al., 2016), *B. litoris,* isolated from sea-tidal flat sediment (Lee et al., 2018), *B. saxobsidens,* isolated from an archaeological site in Greece (Urzì et al., 2004), and *B. xanthinilyticus,* isolated from a marble sample collected from the Bulla Regia monument in Tunisia (Hezbri et al., 2018).

The integration of genomic information into microbial systematics (Klenk and Göker, 2010) along with physiological and chemotaxonomic parameters enables a reliable classification of prokaryotes (Chun et al., 2018). Laboratory-based DNA–DNA hybridisation methods have already been routinely replaced by overall genome relatedness indices (OGRI; Chun et al., 2018) of which digital DNA:DNA hybridization was shown to be the best-performing method (Meier-Kolthoff et al., 2013a). The use of *in silico* analysis for chemotaxonomic traits is now being introduced to replace characteristics traditionally determined in the laboratory (Amaral et al., 2014; Fotedar et al., 2020; Lawson et al., 2020) with the dual goal of both increasing the speed of the description of taxa and the accuracy and consistency of taxonomic reports (Chun and Rainey, 2014; Whitman, 2015).

Genomics has already successfully been applied in the taxonomic rearrangement of *Geodermatophilaceae* (Montero-Calasanz et al., 2017) although other research studies based on genomic data are still scarce in the family. In particular, only two papers were published so far about the genus *Blastococcus* including genomic and proteomic mining of *B. saxobsidens* DD2 and genome mining of *B. atacamensis* which revealed numerous genes involved in stress response and adaptation to harsh habitats (Chouaia et al., 2012; Sghaier et al., 2016) apart from an exceptional potential to produce novel natural compounds (Castro et al., 2018).

Based on genome-scale data and phenotypic evidence according to the principles of phylogenetic systematics (Hennig, 1965; Wiley and Lieberman, 2011), this study characterises four novel species in the genus *Blastococcus,* introduces the new genera *Trujillonella, Pleomorpha,* and *Goekera* in *Geodermatophilaceae* and reclassifies *Blastococcus endophyticus* as *Trujillonella endophytica* comb. nov., *Geodermatophilus daqingensis* as *Pleomorpha daqingensis* comb. nov. and *Modestobacter deserti* as *Goekera deserti* comb. nov., all representing type species of the new genera. Accordingly, we also propose emended descriptions within *Blastococcus*. Additionally, we emphasise how genomics could effectively replace and/or complement some of the phenotypic analyses routinely carried out in microbial systematics.

## Materials and methods

### Isolation

Strains AT 7-1^T^, AT 7–8, AT 7-14^T^, and AT 7(-2)-11^T^ were isolated during a screening of microorganisms from soil samples from the Atacama Desert (Chile) in the 90s and deposited by Prof. Fred A. Rainey (University of Alaska Anchorage, United States) at Leibniz Institute DSMZ. On the other hand, strain G1S^T^ was isolated by Eppard et al. (1996) from leachate of a landfill in Vancouver (Canada). All strains were subsequently accessed in the DSMZ open collection as DSM 44268^T^, DSM 44269, DSM 44270^T^, DSM 44272^T^, and DSM 44205^T^, respectively.

### Genotypic analysis

Genomic DNA extraction, PCR-mediated amplification of the 16S rRNA gene, and purification of the PCR product were carried out as previously described by Rainey et al. (1996). For genome sequencing, strains were cultivated in GYM *Streptomyces* broth (DSMZ medium 65, pH 6.8 ± 0.2; https://www.dsmz.de/collection/catalogue/microorganisms/culture-technology/list-of-media-for-microorganisms) at 28°C and genomic DNA was extracted by using JetFlex™ Genomic DNA Purification Kit (Thermo Fisher Scientific) following the manufacturer’s instructions. The project information is available through the Genomes Online Database (Mukherjee et al., 2017). The draught genomes were generated at the DOE Joint Genome Institute (JGI) as part of the Genomic Encyclopaedia of Archaeal and Bacterial Type Strains, Phase II (KMG-II): “From individual species to whole genera” (Kyrpides et al., 2014) and ACTINO 1000: “Exploiting the genomes of the *Actinobacteria*: plant growth promoters and producers of natural products and energy relevant enzymes united in a taxonomically unresolved phylum,” following the same protocol as in Nouioui et al. (2017). All genomes were annotated using the DOE-JGI annotation pipeline (Huntemann et al., 2015; Chen et al., 2016) and released through the Integrated Microbial Genomes system (Chen et al., 2017). In addition, genomes were uploaded to RAST (Aziz et al., 2008; Brettin et al., 2015) and analysed through the SEED viewer (Overbeek et al., 2014). CRISPRFinder (Grissa et al., 2007) was used to identify CRISPR elements. Further details about sequencing projects are summarised in the Supplementary Table S1.

Phylogenetic analysis of the 16S rRNA gene sequences from the type strains of all species with validated names in *Geodermatophilaceae*, as well as those from *Antricoccus suffuscus* DSM 100065^T^, *Cryptosporangium arvum* DSM 44712^T^, *Cryptosporangium aurantiacum* DSM 46144^T^, *Epidermidibacterium keratini* JCM 31644^T^, *Longivirga aurantiaca* NBRC 112237^T^ and *Sporichthya polymorpha* DSM 43042^T^ for use as outgroup, was conducted as previously described (Göker et al., 2011; Montero-Calasanz et al., 2014). Pairwise 16S rRNA gene sequence similarities were calculated as recommended by Meier-Kolthoff et al. (2013b) to determine strains with ≥99.0% similarity, between which (digital) DNA:DNA hybridization experiments were conducted for clarifying species affiliation. Genome-scale phylogenies were inferred from the available *Geodermatophilaceae* (and outgroup) whole proteome sequences using the high-throughput version (Meier-Kolthoff et al., 2014a) of the Genome BLAST Distance Phylogeny (GBDP) approach (Auch et al., 2010) in conjunction with FastME (Lefort et al., 2015). The tree was rooted according to Nouioui et al. (2018). The entire approach is implemented in the Type (Strain) Genome Server (TYGS; Meier-Kolthoff and Göker, 2019; Meier-Kolthoff et al., 2022). The GBDP tree restricted to the well-supported branches (≥95% pseudo-bootstrap support) was used as a backbone constraint in a subsequent 16S rRNA gene sequence analysis to integrate information from genome-scale data (Hahnke et al., 2016). Digital DNA:DNA hybridisations were conducted using the recommended settings of the Genome-To-Genome Distance Calculator (GGDC) version 3.0 (Meier-Kolthoff et al., 2013a) as implemented in the TYGS (Meier-Kolthoff and Göker, 2019; Meier-Kolthoff et al., 2022). The G + C content was calculated from the genome sequences as described by Meier-Kolthoff et al. (2014b). The genomic homogeneity of strains DSM 44268^T^ and DSM 44269 was examined by ribotyping and matrix-assisted laser-desorption/ionisation time-of-flight (MALDI-TOF) mass spectra (MS). Automated ribotyping of PvuII-digested samples was carried out using the RiboPrinter microbial characterisation system (Qualicon, DuPont) as described in Bruce (1996) and Stackebrandt et al. (2002). MALDI-TOF analysis was performed according to Tóth et al. (2008).

### Phenotypic analysis

#### Morphological and physiological tests

Morphological features of colonies were examined by stereomicroscope on GYM *Streptomyces* agar at 28°C and observed periodically during 7 days. Bacterial cell morphology and motility was determined by phase contrast microscopy with a magnification of 100x and with a field-emission scanning electron microscope (FE-SEM Merlin, Zeiss, Germany). Catalase production was tested on slides with the addition of some drops of 3% H_2_O_2_ solution into a loop of bacterial biomass. It was considered positive when the production of bubbles was observed after mixing. Oxidase activity was determined by obtaining blue-purple colour after addition of some drops of 1% (w/v) solution of *N,N,N′,N′*-tetramethyl-*p*-phenylenediamine (Sigma-Aldrich) to a loop of bacterial biomass spread on filter paper. Gram reaction was performed according to the procedure developed by Gregersen (1978). API ZYM system (bioMérieux) was used to determine enzymatic activities according to the manufacturer’s protocol at 28°C after 24 h incubation. Homogeneous bacterial suspensions of strains DSM 44205^T^, DSM 44268^T^, DSM 44269, DSM 44270^T^, and DSM 44272^T^ along with the reference type strains in the genus *Blastococcus* were prepared in viscous inoculating fluid (IF C) at 86% T (Transmittance) for *B. atacamensis* P6^T^, 83% T for *B. capsensis* DSM 46835^T^, 89% T for DSM 44270^T^*, B. colisei* DSM 46837^T^, and *B. xanthinilyticus* DSM 46842^T^, 90% T for strains DSM 44268^T^, DSM 44269, and DSM 44205^T^, 91% T for DSM 44272^T^, and 95% T for all the others reference strains. Then those were inoculated in GEN III Microplates per duplicate and incubated for 10 days at 28°C in an Omnilog device (BIOLOG Inc., Hayward, CA, United States). Data were analysed using the *opm* package R v.1.3.72 (Vaas et al., 2012, 2013). Reactions that reflected different behaviour between replicates were regarded as ambiguous. Temperature ranges were evaluated on GYM *Streptomyces* medium for 15 days at 4, 10, 15, 20, 25, 30, 37, 40 and 45°C. pH ranges were determined using modified ISP2 medium (Montero-Calasanz et al., 2015) from 4.5 to 12.5 in increments of 0.5 pH unit adjusting with NaOH or HCl since the use of a buffer system inhibited the growth of strains (Montero-Calasanz et al., 2014). pH of solidified plates was measured before inoculation and when recording results (15 days post-inoculation). Hydrolysis of specific substrates such as tyrosine (Gordon and Smith, 1955), casein, starch, xanthine as well as hypoxanthine was also tested as outlined by Montero-Calasanz et al. (2015).

#### Chemotaxonomic tests

For chemotaxonomic analysis, strains were grown in GYM *Streptomyces* broth with shaking (120 rpm) at 28°C for 7 days. Bacterial biomass was collected and subsequently freeze dried. Extraction of whole-cell amino acids and sugars was carried out as developed by Lechevalier and Lechevalier (1970) followed by thin layer chromatography (TLC) analysis and identification (Staneck and Roberts, 1974). Analysis of peptidoglycan hydrolysates (6N HCl, 100°C for 16 h) was performed on cellulose TLC plates as described by Schleifer and Kandler (1972). Menaquinones (MK) analyses (Kroppenstedt, 1982) were accomplished as indicated by Collins et al. (1977) following the identification of MK by high-performance liquid chromatography (HPLC; Kroppenstedt, 1982). Polar lipids were extracted and identified by 2D TLC as outlined by Minnikin et al. (1984) with modifications proposed by Kroppenstedt and Goodfellow (2006). Dragendorff’s reagent (Merck Millipore, 102035) was additionally sprayed to identify choline-containing lipids (Tindall, 1990). For fatty acid analysis, cell biomass grown on PYGV (DSMZ 621) agar plates for 16 days at 20°C was harvested and fatty acid methyl esters were extracted according to Sasser (2001). Microbial Identification System (MIDI) Sherlock Version 6.1 (method TSBA40, ACTIN6 database) was used for performing data analysis. All the chemotaxonomic analyses for strains were carried out under standardised conditions.

## Results and discussion

### Basic genome statistics

Standard draught genomes were obtained for strains DSM 44205^T^, DSM 44268^T^, DSM 44270^T^, and DSM 44272^T^ and type strains of species *B. aggregatus, B. endophyticus* and *B. xanthinilyticus.* An improved-high-quality genome draught according to GOLD sequencing quality standards (Mukherjee et al., 2017) was also obtained for both *B. colisei* DSM 46837^T^ and *B. saxobsidens* DSM 44509^T^ (Supplementary Table S1). The genome sizes ranged from 4.0 Mbp (strain DSM 44205^T^) to 5.1 Mbp (strains DSM 44272^T^ and DSM 46837^T^), with an average genome size for all sequenced *Blastococcus* strains of 4.6 ± 0.4 Mbp (Supplementary Table S2). The genomic G + C content of the sole chromosome in the type strains varied between 72.5–74.6%. Deviations from the published G + C contents of reference strains were all below the 1% threshold determined within species (Meier-Kolthoff et al., 2014b). In accordance with the genome sizes, the number of protein-coding genes ranged from 3,939 for strain DSM 44205^T^ to 5,007 for strain DSM 44272^T^, not observing *a priori* any association between genome size and habitat of isolation. Both tRNA and rRNA genes represented between 1.3% (47 tRNA and five rRNA genes) in *B. xanthinilyticus* DSM 46842^T^ and 2.2% (70 tRNA and nine rRNA genes) in DSM 44205^T^ of the whole genome sequences. A single copy of the 16S rRNA gene was identified for strains DSM 44270^T^, DSM 44272^T^, *B. aggregatus* DSM 4725^T^, and *B. xanthinilyticus* DSM 46842^T^. Three almost identical 16S rRNA genes copies were annotated in the genome sequences of strain DSM 44205^T^, *B. colisei* DSM 46837^T^, *B. endophyticus* DSM 45413^T^, and *B. saxobsidens* DSM 44509^T^, and four almost identical ones in the genome sequence of strain DSM 44268^T^ (intragenomic heterogeneity of 16S rRNA genes is below the 1% level for species delimitation; Meier-Kolthoff et al., 2013a). Previous studies already reported low copy numbers (2–4) for slow-growing actinobacteria (Sun et al., 2013; Vetrovsky and Baldrian, 2013) isolated from oligotrophic environments (Klappenbach et al., 2000). The number of rRNA operon copies is suggested to be related to the life strategy of bacteria and how quickly they respond to favourable changes in environmental conditions and nutrient availability (Klappenbach et al., 2000). Hence, rRNA copy number appears to be correlated with growth speed. The number of identified tRNAs ranged from 47 to 49 in all the *Blastococcus* representatives except in the genome sequence of strain DSM 44205^T^ that contained 70 tRNAs. The 20 standard tRNA genes were present in all genomes but in a different distribution. For example, four tRNA-Arg gene copies (six gene copies for strain DSM 44205^T^) versus one tRNA-Tyr gene copy. Such uneven distribution supports the co-adaptation between tRNA abundance and specific codon usage of each organism as a higher ratio of optimal codons may facilitate the transcription efficiency (Du et al., 2017). Out of the 70 (DSM 44205^T^) and 48 (*B. endophyticus*) annotated tRNAs, seven (DSM 44205^T^) and one (*B. endophyticus*), respectively, were identified as pseudogene(s). The latter are known to be dysfunctional in translation but to be potentially involved in modulating other processes such as antibiotic and cell wall biosynthesis and gene expression (Rogers et al., 2012). One copy of nonsense suppressor tRNA (tRNA amber gene) was also identified in the repertoires of DSM 44270^T^, DSM 44272^T^, and *B. colisei,* which could prevent the premature termination of translation caused by nonsense mutations (Ko et al., 2013). The number of pseudogenes was within the expected values for free-living prokaryotes (0%–5%; Liu et al., 2004) and varied from zero (for example: DSM 44205^T^, DSM 44272^T^, *B. endophyticus, B. xanthinilyticus* or *B. atacamensis*, amongst others) to 123 in *B. colisei* DSM 46837^T^ (2.5%). Each genome contained a largely unique set of pseudogenes, with over half of those annotated as “hypothetical” proteins, suggesting that pseudogenes in *Blastococcus* are formed and eliminated relatively rapidly as previously reported by Kuo and Ochman (2010) for *Salmonella* genomes. In addition, the number of paralogous genes varied from 437 (11%) in strain DSM 44205^T^ to 1,601 (32%) in *B. colisei* DSM 46837^T^, the signal peptide percentage was 3.0%–5.8% whilst the percentage of transmembrane proteins ranged from 19.7% to 24.7%. According to CRISPRFinder, the number of “questionable” Clustered Regularly Interspaced Short Palindromic Repeats (CRISPR) ranged from zero to seven (Supplementary Table S2). Additional analyses to test the genetic environment often associated with CRISPR structures should nevertheless be carried out to confirm the presence of a naturally occurring genome editing system in *Blastococcus* representatives.

The average percentage of genes with a predicted function in the Clusters of Orthologous Groups (COGs) database was 63.3 ± 2.3% (ranged from 2,558 to 3,117 genes; Supplementary Table S2). As expected, functional COG profiles of *Blastococcus* strains are similar (Supplementary Figure S1; Supplementary Table S3).

### Phylogenetic analysis

**Figure 1:**
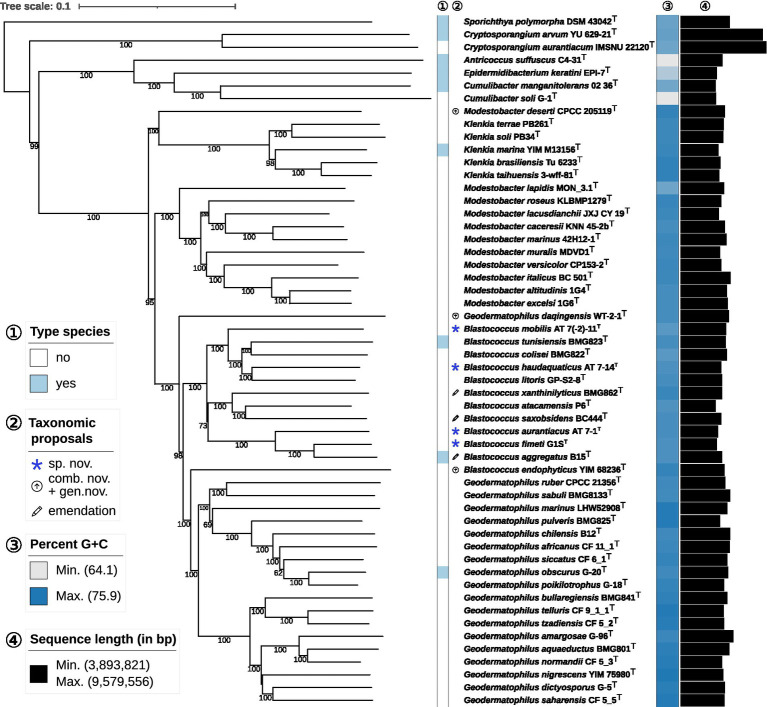
Phylogenomic tree inferred with GBDP. The tree was inferred with FastME from GBDP distances calculated from whole proteomes. The numbers above branches are GBDP pseudo-bootstrap support values from 100 replications. Tip colours indicate genome sizes and the exact G + C content as calculated from the genome sequences (see the embedded legend for details). *A., Antricoccus*; *B., Blastococcus*; *C., Cryptosporangium; G., Geodermatophilus; K., Klenkia; M., Modestobacter; S., Sporichthya.*

The phylogenetic tree inferred by whole proteomes of the sequenced type strains and strains under study revealed the affiliation of DSM 44268^T^, DSM 44270^T^, DSM 44272^T^ and DSM 44205^T^ to the genus *Blastococcus* (Figure 1). The subtree containing the genera *Geodermatophilus, Modestobacter* and *Blastococcus* was not monophyletic which was caused by three misclassified species. First, *B. endophyticus* DSM 45413^T^ appeared with maximum support as a sister group of *Geodermatophilus* and was not phylogenetically placed within the maximally supported subtree harbouring all the other *Blastococcus* type strains. Second, *G. daqingensis* DSM 104001^T^ represented a maximally supported sister group to the large subtree comprising all other *Geodermatophilus* and *Blastococcus* type strains. Third, *M. deserti* CPCC 205119^T^ formed a deeply-branching and maximally supported sister group of *Klenkia*.

**Figure 2:**
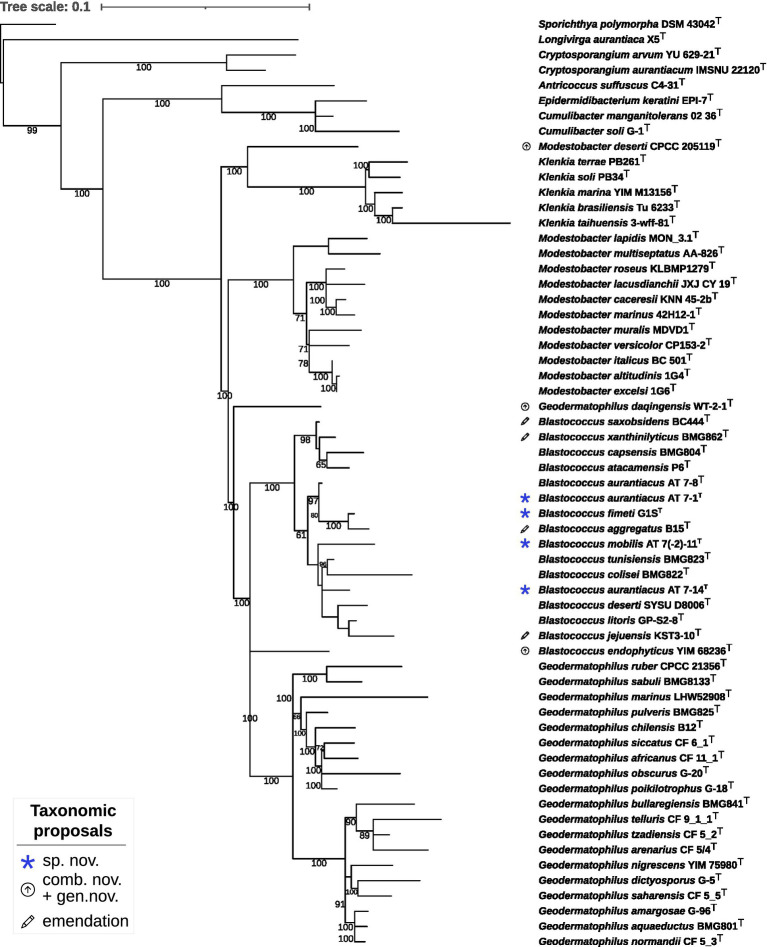
Maximum likelihood phylogenetic tree inferred from 16S rRNA gene sequences, showing the phylogenetic position of the strains DSM 44205^T^, DSM 44268^T^, DSM 44269, DSM 44270^T^ and DSM 44272^T^ relative to the type strains within *Geodermatophilaceae*. The branches are scaled in terms of the expected number of substitutions per site (see size bar). Support values from maximum-likelihood constrained bootstrapping are shown above the branches if equal to or larger than 60%.

The constrained 16S rRNA phylogenetic tree (Figure 2) based on gene sequences of all validly published species in the family and strains under study, including strain DSM 44269, confirmed the observations from the proteome-based GBDP analysis. Moreover, strains DSM 44268^T^ and DSM 44269 were placed with high support in a clade together with strains DSM 44205^T^ and *B. aggregatus* DSM 4725^T^.

The 16S rRNA gene sequence similarity values between (i) DSM 44268^T^ and DSM 44269, (ii) between DSM 44205^T^, DSM 44270^T^ and *G. aggregatus* DSM 4725^T^, and, (iii) between DSM 44270^T^ and *B. litoris* GP-S2-8^T^, ranged above the *Actinobacteria*-specific 16S rRNA threshold of 99.0% (accepting an error probability of 1%; Meier-Kolthoff et al., 2013b).

It was thus necessary to investigate the species status *via* digital DDH (dDDH). The dDDH values between strain DSM 44268^T^ and strains DSM 44205^T^ (30.8%), DSM 44270^T^ (23.8%), and *B. aggregatus* DSM 4725^T^ (30.3%) and between *B. litoris* GP-S2-8^T^ and strains DSM 44270^T^ (26.9%) resulted in values below the 70% threshold throughout, thus indicating distinct species (Wayne et al., 1987).

In addition, the MALDI-TOF dendrogram (Supplementary Figure S2) confirmed the affiliation of strains DSM 44268^T^ and DSM 44269 to the same species showing a 100% similarity regarding mass spectra patterns of proteins and separated from those under study at a distance level > 900 arbitrary units. The riboprinting patterns of the DSM 44268^T^ and DSM 44269 isolates were also highly similar (98.3% similarity; Supplementary Figure S3) but differentiated them as clonal variants of each other.

### Phenotypic-genotypic correlations

#### Morphology and physiology

All target strains were aerobic, Gram-reaction positive and showed coccoid cells with a tendency to form aggregates as previously described by Urzì et al. (2004) for the genus *Blastococcus* (Supplementary Figure S4). In addition, under studied culture conditions, reproduction by budding was predominant although bacterial binary fission was also observed (Supplementary Figure S4). It was already indicated for strain DSM 44205^T^ by Eppard et al. (1996). Effectively, the presence of the cell division protein FtsZ and related genes such as the actin-like protein MreB were annotated in all genomes but no known mechanisms involved in division *via* budding were identified (Supplementary Table S4). Whilst FtsZ is an essential structural homologue of the eukaryotic cytoskeletal element tubulin (Lowe and Amos, 1998) and involved in forming a cytokinetic ring at the division site (Wang and Lutkenhaus, 1993), MreB is mostly essential for cell elongation and maintenance of the cell (Rivas-Marin et al., 2020). Mechanisms of bacterial division by budding are still poorly understood in evolutionary cell biology (Rivas-Marin et al., 2020). *Geodermatophilaceae* representatives are described as aerobic bacteria (Montero-Calasanz, 2020b). Nevertheless, some microaerophilic representatives isolated from the oxic/anoxic interface of marine sediments were also reported (Ahrens and Moll, 1970). To date no anaerobic isolate was described, although the presence of reductases using anaerobic terminal electron acceptors such as arsenate, ferredoxin or flavodoxins as well as a repertoire of genes involved in the fermentation of propanoate, butanoate and pyruvate, amongst others, in the *Blastococcus* genomes could indicate that anaerobic respiration may be possible. It was already suggested by Sghaier et al. (2016).

*In vivo* motility was not observed in any of the studied strains. The presence of homologues involved in flagellar biosynthesis and function and chemotaxis in all the genome sequences, nevertheless, suggests that *Blastococcus* strains may be flagellated chemotactic bacteria and motility may have been influenced by culture conditions. In fact, Ahrens and Moll (1970) suggested that the motile rod stage in *B. aggregatus* could be favoured under microaerophilic conditions. Later studies carried out by Holt and Chaubal (1997) for *Salmonella pullorum* strains, confirmed that flagellar synthesis is affected by agar concentration, carbohydrate concentration and type, and incubation temperature. The presence of flagellar gene clusters is consistent with observed motility by type strains of *B. jejuensis* and *B. saxobsidens* (Urzì et al., 2004; Lee 2006) and other strains in the family (Montero-Calasanz et al., 2017). In fact, flagellin synthesis was identified as the most highly expressed protein in the proteogenome of *Blastococcus* sp. DD2 (Sghaier et al., 2016). The core set of flagellar structural genes for *Geodermatophilaceae* representatives seems to be largely consistent with that described by Liu and Ochman (2007) for the bacterial domain. Twenty-eight different genes comprise specific proteins that form the filament (*fliC*, two copies for strain DSM 44205^T^, DSM 44270^T^, and *B. xanthinilyticus*), the hook-filament junction (*flgL* and *flgK*), the hook (*flgE*), the rod (*flgB* and *flgC*), the MS ring (*fliF*; absent in DSM 44272^T^), the C ring (*fliG, fliM* and *fliN*), the motor (*motA* and *motB*), and the export apparatus (*flhA, flhB, fliP, fliQ,* and *fliR*). In addition, *flgD* encoding for the hook-capping protein, which is required for flagellar assembly (Liu and Ochman, 2007), and other broadly distributed flagellar structural genes such as *fliD* (filament cap), *fliE, fliK* and *fliH* (hook) as well as, *fliZ, fliS* and *fliO* are also part of the core set in *Geodermatophilaceae* (Supplementary Table S4). The L and P ring proteins *FlgH* and *FlhI* are absent in Gram-positive bacteria. Chemotaxis, on the other hand, may be controlled by the universal two-component system. Methyl-accepting chemotaxis proteins (MCP) would control autophosphorylation of a cytoplasmic histidine kinase (*CheA*), *via* a coupling protein (*CheW*), which would activate the response regulator *CheY* and, consequently, interact with the switch mechanism in the flagellar motor (Szurmant and Ordal, 2004). Surprisingly, a dedicated ribose-binding protein (ribose transport system substrate-binding protein, *RbsB*) was annotated in the genome sequences of DSM 44205^T^, DSM 44268^T^, and DSM 44270^T^ suggesting a key role of ribose as chemoeffector in those strains at low concentrations (Galloway and Furlong, 1977).

In addition, *tad* (tight adherence) genes were unambiguously annotated in all the studied genomes (Supplementary Table S4). The *tad* genes encode the machinery required for the assembly of adhesive *Flp* (fimbrial low-molecular-weight protein) pili (Tomich et al., 2007). Those are essential for biofilm formation, colonisation, DNA transfer and pathogenesis and have implications in functions such as phage binding, rough colony morphology, and twitching motility (Proft and Baker, 2009). The presence of Tad complexes in *Geodermatophilaceae* genomes could be important for successful colonisation of altered stones and patina formation (Eppard et al., 1996; Urzì et al., 2001). Howbeit, the presence and abundance of *tad* genes varied amongst strains suggesting that strains may present different adherence-related phenotypes as previously demonstrated by Kachlany et al. (2000) and Perez et al. (2006).

Production of spores was not observed in any of the isolates under the given culture conditions. Nevertheless, the unambiguous presence of single copies of *SsgA*-like proteins (SALPs) in all studied genomes suggested that sporulation may be a typical feature previously overlooked in *Blastococcus* spp. and that, similar to *Geodermatophilus* spp., the presence of SALPs may be affected by growth conditions (Ishiguro and Wolfe, 1970). SALPs occur exclusively in morphologically complex actinomycetes where they play an important role in morphogenesis and control of cell division (Jakimowics and van Wezel, 2012) and are essential for sporulation (van Wezel et al., 2000). Furthermore, the presence of single SALPs (presumably and invariably *SsgB*) in *Blastococcus saxobsidens* DD2 and other *Geodermatophilaceae* representatives was already described by Girard et al. (2013) and is suggested as a link between the number of SALPs and the complexity of the developmental process (Traag and van Wezel, 2008), suggesting that these actinomycetes would produce single spores.

Colonies were opaque with a moist surface and regular margin and varied from coral (strain DSM 44268^T^ and DSM 44205^T^) to brownish-orange in colour (Strains DSM 44270^T^ and DSM 44272^T^ and DSM 44269). Accordingly, phytoene synthase (*crtB;* EC 2.51.32) and phytoene desaturase (*crtI*; 4 step enzyme; EC 1.3.99.28, EC 1.3.99.29, EC 11.3.99.31), the key enzymes involved in the biosynthesis of the colourful carotenoids ζ-carotene, neurosporene, and lycopene (Paniagua-Michel et al., 2012) were identified in all the genomes. Yet, the putative operon mentioned by Sghaier et al. (2016) for *B*. sp. DD2 consisting of *hopC* [squalene-associated FAD-dependent desaturase (fragment)]*, ispA* (generaylgeranyl pyrophosphate synthase)*, shc* (squalene-hopene cyclise), *hpnH* (hopanoid biosynthetic associated radical SAM protein HpnH), was not identified in these genomes, except for crtB and ilvC (ketol-acid reductoisomerase; Supplementary Table S4).

Strains grew well on GYM *Streptomyces* and PYGV agar media. They tolerated temperature ranging from 15°C to 37°C, except strain DSM 44269, which only tolerated a more restricted range from 20°C to 37°C. All strains showed optimal growth at 25°C–30°C. In addition, growth was observed at pH 6.0–12.0 for strains DSM 44268^T^ and DSM 44269, at pH 5.0–11.5 for strain DSM 44205^T^, at 5.0–11.0 for strain DSM 44270^T^, and at 6.5–11.0 for strain DSM 44272^T^. All strains had an optimal pH range from 6.5 to 8.0. Regarding tolerance to salt, strains DSM 44269 and DSM 44272^T^ were the least tolerant (0%–1% NaCl, although at 4% NaCl the Biolog system determined an ambiguous result for DSM 44272^T^), followed by both DSM 44268^T^ and DSM 44205^T^ tolerating 0%–4% NaCl, and strain DSM 44270^T^, which tolerated up to 8% NaCl. A summary of selected differential phenotypic characteristics is presented in Table 1 (for an overview of phenotypic profiles in *Blastococcus* see Supplementary Figure S5; Supplementary Table S5). Details about core metabolism will be provided later on in this manuscript.

**Table 1:** Phenotypic characteristics of strains DSM 44205^T^, DSM 44268^T^, DSM 44269, DSM 44270^T^ and DSM 44272^T^ in comparison with the validly named species in the genus *Blastococcus.*

Ch aracteristics	1	2	3	4	5	6	7	8	9	10	11	12	13
Motility	−	−	−	−	+	+	−	−	−		+	+	−
Oxidation of: Stachyose	+/−	+	+	+/−	+/−	+	+/−	−	+/−	+	+	+	−
α-D-Lactose	−	+	+	−	−	+	+	+	+	−	−	+	+
D-Melibiose	+/−	+/−	+	+/−	−	+	−	+	+/−	+/−	+/−	+	+/−
*N*-Acetyl-D-Galactosamine	−	+	+/−	−	−	+/−	+/−	+/−	+/−	+/−	−	+/−	+/−
D-Mannose	−	−	+	+/−	+	+/−	−	+	−	+/−	+/−	+	+
L-Rhamnose	−	−	−	−	−	+/−	−	+/−	+	+/−	−	+	−
L-Aspartic acid	+/−	+/−	−	+	+/−	−	−	+	+/−	−	+/−	+/−	−
Quinic acid	−	−	+/−	−	+	+/−	+	+/−	+	+/−	+	+/−	−
L-Lactic acid	−	−	−	+	+	−	+/−	−	+/−	−	+	−	−
Citric acid	+	−	+	+	−	+	+/−	+/−	+/−	−	+/−	−	−
α-Keto-Glutaric acid	+	+	+	−	+/−	+	+	+	+	+	+	+	+/−
D-Malic acid	+/−	+	+	−	−	+	+	+	+/−	+	+	−	+
L-Malic acid	−	+	+	+	−	+/−	+/−	+	+/−	+	+	−	−
Polar lipids	DPG, PC, PE, PI	DPG, PC, PE, PG, PI	DPG, PC, PE, PG, PI	DPG, PC, PE, PG, PI	DPG, PC, PE, PG, PI	DPG, PC, PI	DPG, PC, PE, PG, PI	DPG, PC, PE, PG, PI	DPG, PC, PE, PG, PI	DPG, PC, PE, PI	DPG, PC, PE, PI	DPG, PC, PE, PI	DPG, PC, PE, PG, PI
Sugars	Ribose Xylose Arabinose Mannose Glucose	Rhamnose Ribose Arabinose Mannose Glucose Galactose	Ribose Arabinose Mannose Glucose Galactose	Ribose Mannose Glucose Galactose	Rhamnose Ribose Arabinose Glucose Galactose	Ribose Arabinose Mannose Glucose	Ribose Glucose Galactose	Rhamnose Ribose Glucose	Ribose Glucose Galactose	Rhamnose Xylose Ribose Mannose Glucose	Rhamnose Ribose Arabinose Mannose Glucose	Ribose Mannose Glucose Galactose	Ribose Glucose Galactose
Menaquinones^a#^	MK-9(H_4_) MK-8(H_4_)	MK-9(H_4_) MK-9(H_0_) MK-10(H_4_)	MK-9(H_4_) MK-9(H_2_) MK-9(H_0_) MK-10(H_4_)	MK-9(H_4_) MK-9(H_2_) MK-8(H_4_) MK-9(H_0_) MK-9(H_6_)	MK-9(H_4_) MK-9(H_2_) MK-9(H_0_)	MK-9(H_4_) MK-8(H_4_)	MK-9(H_4_) MK-9(H_2_)	MK-9(H_4_) MK-9(H_2_) MK-9(H_0_)	MK-9(H_4_) MK-8(H_4_) MK-9(H_0_)	MK-9(H_4_) MK-8(H_4_) MK-9(H_0_) MK-8(H_0_)	MK-9(H_4_) MK-9(H_0_)	MK-9(H_4_) MK-9(H_2_) MK-8(H_4_) MK-9(H_0_) MK-9(H_6_)	MK-9(H_4_) MK-9(H_2_) MK-8(H_4_)
Fatty acids^b^	iso-C_16:0_ anteiso-C_17:0_ anteiso-C_17:1_ iso-C_16:1_ C_18:1_*ω*9c	C_18:1_*ω*9c iso-C_16:0_ iso-C_15:0_ iso-C_16:1_	C_18:1_*ω*9c iso-C_16:0_ iso-C_15:0_ C_16:1_*ω*7c C_16:0_	iso-C_16:0_ C_17:1_*ω*8c iso-C_16:1_ iso-C_15:0_ C_18:1_*ω*9c	iso-C_16:0_ C_18:1_*ω*9c C_17:1_*ω*8c iso-C_15:0_ iso-C_16:1_	iso-C_16:0_ iso-C_16:1_ C_18:1_*ω*9c	iso-C_16:0_ iso-C_16:1_ iso-C_15:0_ 9-methyl-C_16:0_	C_17:1_*ω*8c C_16:1_*ω*7c iso-C_15:0_ iso-C_16:0_ iso-C_16:1_	iso-C_16:0_ iso-C_16:1_ C_17:1_*ω*8c C_18:1_*ω*9c iso-C_15:0_	iso-C_16:0_ iso-C_15:0_ iso-C_16:1_ C_16:1_*ω*7c	iso-C_16:0_ C_18:1_*ω*9c C_17:1_*ω*8c iso-C15:0	iso-C_16:0_ C_17:1_ω8c iso-C_16:1_ iso-C_15:0_	iso-C_16:0_ C_17:1_ *ω*8c iso-C_15:0_ C_18:1_ *ω*9c iso-C_16:1_

Strains: 1, strain DSM 44205^T^; 2, strain DSM 44268^T^; 3, strain DSM 44269; 4, strain DSM 44270^T^; 5, strain DSM 44272^T^; 6, *B. aggregatus* DSM 4725^T^; 7, *B. atacamensis* NRRL B-65468^T^; 8, *B. capsensis* DSM 46835^T^; 9, *B. colisei* DSM 46837^T^; 10, *B. endophyticus* DSM 45413^T^; 11, *B. jejuensis* DSM 19597^T^; 12, *B. saxobsidens* DSM 44509^T^; 13, *B. xanthinilyticus* DSM 46842^T^. +, positive reaction; −, negative reaction; +/−, ambiguous; DPG, diphosphatidylglycerol; PE, phosphatidylethanolamine; PG, phosphatidylglycerol; PI, phosphatidylinositol; PC, phosphatidylcholine; MK, menaquinones.

### Chemotaxonomy

Whole-cell hydrolysates of all strains showed *meso*-diaminopimelic (DAP) acid (Cell wall type III; Lechevalier and Lechevalier, 1970) as the diagnostic diamino acid being consistent with the family *Geodermatophilaceae* (Montero-Calasanz, 2020b) and genomic data (presence of *MurE* encoding for UDP-N-acetylmuramoylalanyl-D-glutamate--2,6-diaminopimelate ligase [6.3.2.13] and absence of UDP-N-acetylmuramoyl-L-alanyl-D-glutamate—L-lysine ligase [6.3.2.7]; Supplementary Table S4). In addition, D-alanyl-D-alanine carboxypeptidase [3.4.16.4] was automatically annotated in all *Blastococcus* representative genomes except in *B. atacamensis* P6^T^ and in most *Geodermatophilaceae* representatives. The presence of diaminopimelate epimerase (*dapF*), responsible for the interconversion of the LL- and *meso*- isomers of DAP (Antia et al., 1957), was also annotated in all genomes including in the genome of *Sporichthya polymorpha* DSM 43042^T^, whose cell wall is characterised by containing large amounts of LL-DAP (Trujillo and Normand, 2019). In this way, genomic data could easily be used to distinguish taxa with DAP-type peptidoglycan from those with Lys-type pepdioglycan but fail to differentiate those incorporating LL-DAP (e.g., *Sporichthyales*) from those incorporating *meso*-DAP (e.g., *Geodermatophilales*). Although the evolutionary explanation for the incorporation of one or another stereoisomer is still unclear, studies in *E. coli* suggest that the differentiation of DAP stereoisomers might be related to dimerization of the DAP epimerase (Mengin-Lecreulx et al., 1988; Hor et al., 2013).

As suggested by Hezbri et al. (2016) for the emendation of the genus *Blastococcus*, whole-cell sugar analysis displayed a basic pattern consisting of ribose, glucose, and mannose. Additionally, galactose was identified in all strains under study except in the profile of strain DSM 44205^T^. Similarly, arabinose was found as part of the sugar cell wall composition in all studied strains excluding strain DSM 44270^T^. Xylose was detected in strain DSM 44205^T^.

The predominant menaquinone (MK) for strains DSM 44205^T^, DSM 44268^T^, DSM 44269, DSM 44270^T^, and DSM 44272^T^ was MK-9(H_4_; 75.9%, 92.6%, 56.7%, 78%, and 62.9%, respectively) in agreement with what was described for *Geodermatophilaceae* (Montero-Calasanz et al., 2017). MK-9(H_0_) was however also found in minor levels in the strains isolated from Atacama soils (DSM 44268^T^, 0.7%; DSM 44269, 17.4%; DSM 44270^T^, 7.4%; DSM 44272^T^, 20.2%). Minor amounts of MK-9(H_2_) were also observed in the MK profiles of strains DSM 44269, DSM 44270^T^, and DSM 44272^T^ (1.6, 1.3, and 15.0%, respectively). MK-8(H_4_) was also identified in strains DSM 44205^T^ (5.5%) and DSM 44270^T^ (2.9%). But MK-10(H_4_) was only found in strains DSM 44268^T^ (4.7%) and DSM 44269 (0.6%) and MK-9(H_6_) in strain DSM 44270^T^ (1.1%). The presence of quinones other than MK-9(H_4_) is commonly observed in other representatives in *Geodermatophilaceae* (Montero-Calasanz et al., 2017; Montero-Calasanz, 2020b, 2021), although it is worth mentioning that this is the first report of MK-10(H_4_) in members of the genus *Blastococcus*. The biosynthesis of menaquinones, *via* chorismate derived from Shikimate pathway (genes: *MenA-G*), was confirmed by genomic data in the order *Geodermatophilales*. Curiously, the futalosine pathway, the other major type of menaquinone biosynthetic pathway (Seto et al., 2008) was also annotated in genomes of *Cryptosporangium arvum* and *Crytosporangium aurantiaca* type strains (Supplementary Table S4). *MenJ* encoding for menaquinone-9-*β*-reductase [EC:1.3.99.38] and implicated in menaquinone side chain saturation (Upadhyay et al., 2015) was misidentified by databases as a geranylgeranyl reductase family protein but was verified in all genomes after BLAST using UniProtKB:P9WNY8 (MENJ_MYCTO). Moreover, the presence of 1,4-dihydroxy-2-naphthoate polyprenyltransferase [EC:2.5.1.74] and demethylmenaquinone methyltransferase [EC:2.1.1.163] in all genomes confirmed the potential of *Geodermatophilales* representatives to synthesise MK 8, 9, and 10. Regarding the elongation of isoprenoid side chains, genes *uppS* and *hepST*, encoding for ditrans,polycis-undecaprenyl-diphosphate synthase ((2E,6E)-farnesyl-diphosphate specific) and polyprenyl diphosphate synthase (annotated here as geranylgeranyl pyrophosphate synthase), respectively, were annotated in all genomes. But the length and the degree of saturation of the C-3 isoprenyl side chains were impossible to predict. MKs consist of repeated isoprene subunits and the exact number is determined by the synthase encoded by the particular microbe and a microbe-dependent molecular ruler mechanism that involves bulky amino acid residues blocking the enzyme active sites to stop chain elongation (Han et al., 2015). The inability to predict the isoprenyl chain length of menaquinones was already reported by Baek et al. (2018).

Regarding fatty acid profiles, the five strains qualitatively showed similar patterns to those found in other representatives in *Blastococcus* (Table 1), but the predominant fatty acids significantly varied amongst them. Dominant fatty acids for strains DSM 44268^T^ and DSM 44269 (>5%) were C_18:1_*ω*9c (36.2% and 37.2%, respectively), iso-C_16:0_ (19.3% and 11.3%, respectively), iso-C_15:0_ (6.2% and 8.6%, respectively), and C_16:1_*ω*7c (7.8% and 7.2%, respectively). Additionally, DSM 44268^T^ showed iso-C_16:1_ H (5.4%) and DSM 44269 displayed some minor amounts of C_16:0_ (5.6%) and C_17:1_*ω*8c (6.8%). Those latter fatty acids were also present in DSM 44268^T^ and iso-C_16:1_ H in DSM 44269 but at levels below 5%. On the other hand, strain DSM 44270^T^ had the major fatty acids (>5%) iso-C_16:0_ (32.0%), C_17:1_*ω*8c (27.0%), iso-C_16:1_ H (8.7%), iso-C_15:0_ (7.3%), and C_18:1_*ω*9c (6.0%). A similar composition was observed for strain DSM 44272^T^ although those quantitatively varied as follows: iso-C_16:0_ (24.0%), C_18:1_*ω*9c (23.0%), C_17:1_*ω*8c (13.7%), iso-C_15:0_ (10.8%), and iso-C_16:1_ H (8.1%). Finally, the major fatty acids of strain DSM 44205^T^ (>5%) consisted of iso-C_16:0_ (23.4%), anteiso-C_17:0_ (19.8%), and anteiso-C_17:1_ C (11.9%), iso-C_16:1_ H (11.1%), and C_18:1_*ω*9c (6.7%). The complete gene set (*Fab* cluster) related to fatty acids metabolism (type II fatty acid synthase (FAS II) system) was present in all the studied genomes. Similar to the biosynthesis of MKs, fatty acid synthesis occurs *via* recurring reactions. It is thus not possible to predict just by using genomic data modifications that result in desaturations and/or elongations (López-Lara and Geiger, 2019).

**Figure 3:**
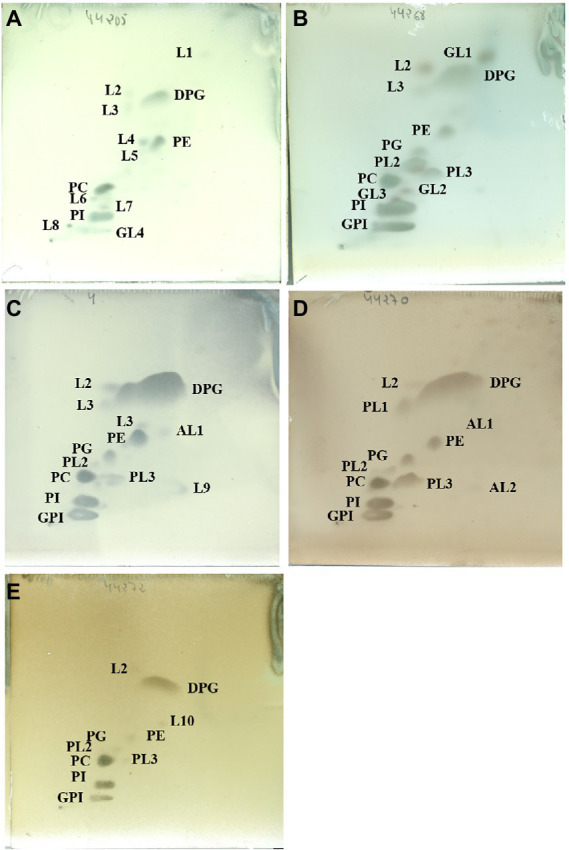
Polar lipids profile of strains DSM 44205^T^**(A)**, DSM 44268^T^ **(B)**, DSM 44269 **(C)**, DSM 44270^T^**(D)** and DSM 44272^T^ **(E)** after separation by two-dimensional TLC using the solvents chloroform:methanol:water (65:25:4; v:v:v) in the first dimension and chloroform:methanol:acetic acid:water (80:12:15:4; v:v:v:v) in the second one. Plates were sprayed with molybdatophosphoric acid (3.5%; Merck™) for detection of the total polar lipids. DPG, diphosphatidylglycerol; PG, phosphatidylglycerol; PE, phosphatidylethanolamine; PI, phosphatidylinositol; PC, phosphatidylcholine; GPI, glycophosphatidylinositol; PL1-3, Phospholipid; GL1-4, glycolipid; AL1-2, aminolipid; L1-10, unidentified lipids. All data are from this study.

The major polar lipids were diphosphatidylglycerol (DPG), phosphatidylethanolamine (PE), phosphatidylcholine (PC), and phosphatidylinositol (PI; Figure 3). It was consistent with those already described by Hezbri et al. (2016). Significant amounts of phosphatidylglycerol (PG) were also identified in all the isolates except in strain DSM 44205^T^. The inconsistent presence of PG was already noted for other species in the family (Montero-Calasanz et al., 2014, 2015, 2017; Hezbri et al., 2015a,b, 2016). Similarly, the characteristic glycophosphoinositol (GPI; annotated as GPL in Hezbri et al., 2016) as well as the reproducible presence of two unidentified polar lipids (PL2 and PL3) was detected in strains DSM 44268^T^, DSM 44269, DSM 44270^T^, DSM 44272^T^ but not in DSM 44205^T^. However, a minor amount of an unidentified glycolipid (GL4) was found in the polar lipids pattern of strain DSM 44205^T^. Three different ones (GL1, GL2, and GL3) were also identified in the profile of strain DSM 44268^T^ but not in strain DSM 44269. In contrast, an unidentified aminolipid (AL1) was displayed by strain DSM 44269 but not by DSM 44268^T^. One having a similar chromatographic mobility was present in strain DSM 44270^T^, too. Genomic data were consistent with those obtained experimentally with some minor discrepancies. In particular, the enzymes phosphatidylcholine synthase and cardiolipin synthase [EC 2.7.8.41] required in the synthesis of PC and cardiolipin (DPG), respectively, were present in all the genomes. As expected, phosphatidylcholine synthase was missing in *Modestobacter* and *Klenkia* type species. CDP-diacylglycerol--glycerol-3-phosphate 3-phosphatidyltransferase [EC 2.7.8.50] involved in the synthesis of phosphatidyl-glycerophosphate, and also in fatty acids metabolism, was present in all species although phosphatidylglycerophosphatase [EC 3.1.3.27] required to the final conversion to PG was just annotated in DSM 44272^T^ and in *B. litoris*. Phosphatidylserine descarboxylase [EC 4.1.1.65] required to synthesise phosphatidylethanolamine was not annotated in *M. multiseptatus*. CDP-diacylglycerol—inositol 3-phosphatidyltransferase [EC 2.7.8.11] needed for the synthesis of PI was annotated in all genomes. Finally, some other enzymes involved in the biosynthesis of other polar lipids were also annotated in the studied genomes suggesting that those may correspond to those minor unidentified lipids shown on the TLC plate.

#### Carbon metabolism

As expected, and in agreement with results determined by GEN III Biolog System (Supplementary Table S5), *Blastococcus* spp. show aerobic microbial respiration potentially driven by glycolysis, the tricarboxylic acid (TCA) cycle, and oxidative phosphorylation. In addition, CO_2_ could be assimilated *via* C4-dicarboxylic acid cycle by phosphoenolpyruvate carboxylase [EC 4.1.1.31], which converts phosphonolpyruvate coming from pyruvate metabolism, glucolysis or gluconeogenesis into oxaloacetate, and is present in all species except DSM 44268^T^ and *B. aggregatus*. A second mechanism to fixate CO_2_ based on the reductive citric acid is also observed in all species (i.e., a molecule of CO_2_ is incorporated along succinyl-CoA, *via* 2-oxoglucarate/2-oxoacid ferredoxin oxidoreductase [EC 1.2.7.3], which in turn will be converted into isocitrate *via* isocitrate dehydrogenase [EC 1.1.1.42], using another molecule of CO_2_). The presence of carbonic anhydrases (CAs, EC 4.2.1.1), which are involved in the catalysis of CO_2_ to bicarbonate and vice versa (Supuran and Capasso, 2017) is found in all the genomes. Finally, the Wood-Ljungdahl pathway, which assimilates CO_2_ into Acetyl-CoA *via* the complex carbon monoxide deshydrogenase/acetyl-CoA synthase (CODH/ACS), may potentially be present. A complete tetrahydrofolate (THF) methyl-branch along with the presence of carbon monoxide dehydrogenases, required for the carbonyl-branch, were annotated in all genomes. These microorganisms could also be able to produce acetate as end product (acetogenesis). But no acetyl-coA synthase [2.3.1.169] homologue was identified in this study. And neither an autotroph nor an acetogen was described in the family so far. Overall, the presence of a number of mechanisms related to chemolithoautotrophy denotes that *Blastococcus* representatives could play a key role in the global carbon cycle. Microorganisms able to efficiently capture and store CO_2_ are prime candidates to be used as biological approaches in the reduction of the atmospheric CO_2_ concentration and as production platforms for a wide range of bioproducts from CO_2_ (Katsyv and Müller, 2020).

Apart from D-glucose as carbon source, genomics data identified other potential carbon sources such as fructose (fructokinase mediated: EC 2.7.1.4); α-galactose (*galk*, galactokinase); D-galactose (*galM*, Aldose 1-epimerase; EC 5.1.3.3); which is absent in DSM 44205^T^; L-gulose (*gnl*, RGN gluconolactonase; EC 3.1.1.17); 1-butanol (alcohol deshydrogenase; EC 1.1.1-), only present in *B. aggregatus*; D-mannose-6P/1P (*manB* phosphomannomutase; EC 5.4.2.8 /*manC* mannose-1-phosphate guanylyltransferase; EC 2.7.7.13), absent in *B. aggregates*; glycine *via* GDC glycine dehydrogenase and glycine hydroxymethyltransferase (*glyA*, EC 2.1.2.1); L-glutamate (*gdhA*, glutamate dehydrogenase; EC 1.4.1.3); L-serine (glycine hydroxymethyltransferase; *glyA*, EC 2.1.2.1), isocitrate (isocitrate dehydrogenase), absent in DSM 44272^T^; formamide (formamidase; EC 3.5.1.49) present in DSM 44205^T^, DSM 44272^T^, and *B. aggregatus*; trehalose (*treS*, maltose α-D-glucosyltransferase; EC 4.99.16 and D-glucose α,α-trehalose phosphorylase; EC 2.4.1.65); L-lactate (*lldD*, L-lactate dehydrogenase; EC 1.1.2.3), just present in *B. endophyticus*; sucrose (*malZ*, α-glucosidase; EC 3.2.1.20); maltose (maltokinase; EC 2.7.1.175), absent in *B. xanthinilyticus*; isomaltose (oligo-1,6-glucosidase), amongst others, that may also be utilised by *Blastococcus* strains. These results supported the presence of a rich carbohydrate metabolism in the genus and were largely in correlation with those showed by GEN III Biolog and API System (See Supplementary Figure S5; Supplementary Tables S4, S5).

Additionally, *Blastoccocus* genomes revealed extracellular enzymes involved in the degradation of complex carbohydrates, which are closely linked to the decay of organic residues, transformation of native soil organic matter, mineralisation of plant nutrients, and soil aggregation (Balezentiene, 2012). For example, *α*-glucosidases [EC 3.2.1.20], involved in the cellulose hydrolysis, were observed in all studied genomes (three copies in *B. xanthinilyticus*); *β*-glucosidases [EC 3.2.1.21] were also annotated in all of them except in *B. colisei* and *B. xanthinilyticus,* having three gene copies in DSM 44205^T^, DSM 44268^T^, and *B. saxobsidens;* Moreover, all studied genomes contained *α*-amylases [EC 3.2.1.1], extracellular enzymes handling the almost complete starch saccharification (Tu and Miles, 1976), but enzymatic activity was not recorded in any of them according to the APIM ZYM system; the presence of glycosidase (glycoside hydrolase; annotated as *α*-amylase by Pfam database) was, nevertheless, only annotated in *B. endophyticus;* Finally, chitinases [EC 3.2.1.14] were annotated in *B. endophyticus* and *B. saxobsidens.* Cellulases (*β*-1,4-endoglucan hydrolase; EC 3.2.1.4) and 1,4- *β*- cellobiohydrolase [EC 3.2.1.91] were absent. On the other hand, lipolytic enzymes are an important group of biotechnologically relevant enzymes presenting a key role in regulating the levels of hydrocarbons in soil (Gupta et al., 2004). Extracellular lipases such as the well-known triacylglycerol acylhydrolase (here as tryacylglycerol lipase, EC 3.1.13) was only annotated in DSM 44268^T^. In addition, lysophospholipases [EC 3.1.1.5] and monoglyceride lipases (EC 3.1.1.23) were present in all the genomes analysed except DSM 44272^T^ and *B. endophyticus*. Polyhydroxyalkanoic acid (PHA) synthases (EC 2.3.1.-), the key enzyme in the biosynthesis of PHAs, a class of aliphatic polyesters that are generally regarded as a carbon and energy reserve material in bacteria and archaea (McCool and Cannon, 2001), were also identified in all the genomes in a number of copies that ranged from one to six with the exception of DSM 44272^T^. Again, large correlations but also discrepancies were observed between genomic and experimental data. For example, the hydrolysis of xanthine by *B. xanthinilyticus,* previously described by Hezbri et al. (2018), was confirmed by the presence of xanthine deshydrogenase but experimentally it could not be proven for strains DSM 44205^T^ and DSM 44268^T^ and *B. endophyticus* although such enzyme was also annotated in their genomes. Similar inconsistencies were also identified for other unambiguously annotated enzymes such as *α*-glucosidase which was not expressed under standardised experimental conditions in strains DSM 44205^T^, DSM 44268^T^, DSM 44270^T^, DSM 44272^T^, and *B. aggregatus* or *β*-glucosidase which was only expressed by *B. endophyticus* and *B. saxobsidens* (for more examples see Supplementary Figure S5; Supplementary Tables S4, S5).

## Final remarks and taxonomic consequences

The integration of genomic information into microbial systematics have proven to be a reproducible, reliable and highly informative approach to reveal phylogenetic relationships amongst prokaryotes (Chun and Rainey, 2014; Chun et al., 2018). Montero-Calasanz et al. (2017) already applied phylogenomics in combination with the identification of diagnostic features in the taxonomic rearrangement of *Geodermatophilaceae.* In light of new genomic information now available, the taxonomic status of the family was revisited in this study. The whole proteome-based phylogenomic tree of the sequenced type strains and strains under study along with phenotypic tests showed with strong support that DSM 44268^T^, DSM 44270^T^, DSM 44272^T^, and DSM 44205^T^ represent novel species within the genus *Blastococcus.* Additionally, the non-monophyly of *Blastococcus, Geodermatophilus* and *Modestobacter* was revealed, i.e., *B. endophyticus, G. daqingensis* and *M. deserti* formed separate lineages within *Geodermatophilaceae*. Based on principles of phylogenetic systematics and applying taxonomic conservatism, we propose the following reclassifications: *Blastococcus endophyticus* as the type species of the new genus *Trujillonella* gen. nov., which the name *Trujillonella endothytica* comb. nov is given; *Geodermatophilus daqingensis* as the type species of the new genus *Pleomorpha* gen. nov., which the name *Pleomorpha daqingensis* comb. nov is given; *Modestobacter deserti* gen. nov., as the type species of the new genus *Goekera* which the name *Goekera deserti* comb. nov is given. Finally, based on the additional taxonomic data now available we propose the emendation of *B. aggregatus, B. jejuensis, B. saxobsidens* and *B. Xanthinilyticus* to include accession numbers for the whole genome sequences, genome size and genomic G + C content and insights. The use of genomic data for the characterisation of novel species in *Geodermatophilaceae* also revealed interesting insights regarding their metabolism and ecology. In particular, the number of 16S rRNA and tRNA gene copies was suggested to be related to their slow growth speed and their life strategy supporting their prevalence in oligotrophic environments such as desert soils and decayed monuments (Neilson et al., 2012; Giongo et al., 2013). In addition, the presence of a repertoire of genes related to flagellum synthesis, chemotaxis, spore production and pilus assembly in all representatives of the family highlighted potential yet unobserved features that would enable us to better understand their metabolism, morphogenesis and ecology. Combining high-throughput phenotype analyses with genomics also supported the uncovering of a rich carbon metabolism in *Blastococcus* spp. Results obtained from GEN III microplates and API System were largely correlated with what was observed in the genomes. In this way, *Blastococcus* spp. were described as aerobic bacteria with the potential to present anaerobiosis and fixate CO_2_ by using four potential pathways and to degrade a range of complex carbohydrates. Such metabolic versatility would support their success to colonise a variety of biotopes and a potential key role in the global carbon cycle and as biological tools to reduce the atmospheric CO2 concentration and produce bioproducts from CO_2._

Even though chemotaxonomic procedures marked a turning point in prokaryotic systematics (Goodfellow et al., 2012), results can be influenced by cultivation conditions (i.e., fatty acid analysis) or by inexperienced staff (i.e., regarding the interpretation of polar lipids patterns on 2-D TLC plates; Sutcliffe et al., 2013). In favour of a revitalization of bacterial taxonomy, *in silico* chemotaxonomic analyses were already used in some studies to complement and, in some cases, replace characteristics traditionally determined in the laboratory (Amaral et al., 2014; Fotedar et al., 2020; Lawson et al., 2020). Here, we attempted to determine *in silico* cell-wall peptidoglycan, respiratory quinone, fatty acids and polar lipid patterns of *Geodermatophilaceae*. Overall, genomic data were consistent with the results obtained from the laboratory. Nevertheless, the amount of information that can be predicted based on genomic data varied depending on the chemotaxonomic trait. For instance, the *in silico* polar lipid analyses were largely in line with those obtained experimentally and allowed us to distinguish at the genus level by the presence/absence of the gene encoding for phosphatidylcholine synthase. On the other hand, the presence of the DAP as the diagnostic diamino acid over L-lysine in the whole-cell hydrolysates was confirmed but the *in silico* procedure failed in differentiating microorganisms incorporating LL-DAP rather than *meso*-DAP (discrimination at order level). Regarding both MK and fatty acids analysis, final products could not be predicted due to the nature of biosynthetic pathways. Nevertheless, sufficient information would, for example, be gathered from Shikimate pathway along with the absence of futalosine pathway to establish certain taxonomic discrimination at order level. The use of genomic data for species delineation and the impractical value of chemotaxonomic data when genomic data are available are widely proven (Nouioui et al., 2018). Nevertheless, as argued by Vandamme and Sutcliffe (2021), knowledge of cellular components can be valuable to establish discriminative thresholds when applied at taxonomic ranks above the species level. However, improvements in analytical methods leading to the use of automated and higher-resolution technologies such as mass spectrometry lipidomic methods (Rashid et al., 2017) should first be introduced and deployed. Our results here proved the use of *in silico* chemotaxonomic analysis as a simple and fast approach to complement phylogenomic results and to provide sufficient resolution to support the affiliation of novel isolates into appropriate taxonomic groups. Its routine application would undoubtedly increase the speed of taxon descriptions and the accuracy and consistency of taxonomic reports.

In summary, the integration of genomics into the systematics of *Geodermatophilaceae* not only allowed for more stable and reliable taxonomic arrangements but also showed the feasibility for a potential replacement of wet-lab chemotaxonomy. Second, it provided deeper insights into the molecular mechanisms behind phenotypic features and revealed potentially overlooked ones that could be key to an understanding of their evolution, ecology and biotechnological potential.

### Description of *Trujillonella* gen. nov.

Tru.jil.lo.nel’la. N.L. fem. n. *Trujillonella,* named in honour of Martha E. Trujillo in recognition of her contributions to microbial systematics, mainly on Actinobacteria, on Bergey’s Manual trust, and as the Editor-in-chief of the International Journal of Systematic and Evolutionary Microbiology.

Cells are aerobic, non-motile, non-spore-forming, Gram-stain positive, catalase-positive and oxidase-negative. Cells occur singly, in pairs or in tetrads, often tending to form aggregates. The peptidoglycan in the cell-wall contains *meso*-diaminopimelic acid. The predominant menaquinone is MK-9(H_4_), with MK-8 and MK-9(H_6_) as minor components. The basic polar lipid profile contains diphosphatidylglycerol, phosphatidylcholine, phosphatidylethanolamine, and phosphatidylinositol. The major fatty acids are iso-C_16:0_, iso-C_15:0_ and C_18:1_*ω*9c. The basic whole-cell sugar pattern includes arabinose and galactose. The genomic G + C content is 71%–72%.

The type species of *Trujillella* is *Trujillella endophytica*, sp. nov.

### Description of *Trujillella endophytica* comb. nov.

*T*. en.do.phy’ti.ca (Gr. pref. *Endo-,* within; Gr. neut. n. *phyton,* plant; L. fem. Adj. suff. –*ica,* adjectival suffix used with the sense of belonging to; N.L. fem. Adj. *endophytica,* within plant, endophytic, pertaining to the isolation from plant tissues).

Basonym: *Blastococcus endophyticus* Zhu et al. (2013) emend. Hezbri et al. (2016).

The properties are as given in the species description by Zhu et al. (2013) and emendation by Hezbri et al. (2016) with the following modification. The genomic G + C content is 74.6%. The genome size is 4.9 Mbp. According to genomic data, anaerobiosis and acetogenesis may occur. A repertoire of genes related to flagellum synthesis, chemotaxis, spore production and pilus assembly were annotated. Four different autotrophic mechanisms including the Wood-Ljungdahl pathway, C4-dicarboxylic acid and reductive citric acid cycles and carbonic anhydrases as well as a range of genes involved in the degradation of complex carbohydrates were also identified.

The accession number for the whole genome sequence of strain DSM 45413^T^ is FOEE00000000.

The type strain YIM 68236^T^ = CCTCC AA 209045^T^ = DSM 45413^T^ = KCTC 19998^T^ was isolated from healthy leaves of *Camptotheca acuminate* collected in Yunnan Province, south-west China.

### Description of *Pleomorpha* gen. nov.

Ple.o.mor’pha. Gr. adv. *Pleon* more; Gr. fem. n. *morphe*, shape or form; N.L. fem. n. *Pleomorpha*, organism showing multiple forms.

Pleiomorphic, motile, spore-forming, aerobic, Gram-stain positive cells. Those occurs singly or associated in aggregates. The peptidoglycan in the cellwall contains *meso*-diaminopimelic acid. The predominant menaquinone is MK-9(H_4_). The basic polar lipid profile contains diphosphatidylglycerol, phosphatidylcholine, phosphatidylglycerol, phosphatidylethanolamine, and phosphatidylinositol. The major fatty acids are iso-C_16:0_ and iso-C_15:0_. The basic whole-cell sugar pattern includes galactose, glucose and xylose. The genomic G + C content is 73%–74%. The type species of *Pleomorpha* is *Pleomorpha daqingensis*, sp. nov.

### Description of *Pleomorpha daqingensis* comb. nov.

*P*. da.qing.en’sis (N.L. masc./fem. Adj. *daqingensis*, pertaining to Daqing city, China, where the type strain was isolated).

Basonym: *Geodermatophilus daqingensis* Wang et al. (2017).

The properties are as given in the species description by Wang et al. (2017) with the following modification. The genomic G + C content is 73.6%. The genome size is 5.4 Mbp. According to genomic data, anaerobiosis and acetogenesis may occur. A repertoire of genes related to flagellum synthesis, chemotaxis, spore production and pilus assembly were annotated. Four different autotrophic mechanisms including the Wood-Ljungdahl pathway, C4-dicarboxylic acid and reductive citric acid cycles and carbonic anhydrases as well as a range of genes involved in the degradation of complex carbohydrates were also identified.

The accession number for the whole genome sequence of strain DSM 104001^T^ is JACBZT000000000.

The type strain WT-2-1^T^ = CGMCC 4.7381^T^ = DSM 104001^T^ was isolated from pretroleum-contaminated soil in Daqing city, China.

### Description of *Goekera* gen. nov.

Goe’ke.ra. N.L. fem. n. Goekera, named in honour of Markus Göker in recognition of his contributions to microbial systematics, including work on *Actinobacteria*, on the List of Prokaryotic Names with Standing in Nomenclature (LPSN), and as a member of the Judicial Commission.

Cells are motile, non-spore-forming, aerobic, Gram-stain positive, catalase and oxidase positive cocci and/short rods. Bud-like structure was observed for some cells. The peptidoglycan in the cell-wall contains *meso*-diaminopimelic acid. The predominant menaquinone is MK-9(H_4_), with MK-8(H_4_) as a minor component. The basic polar lipid profile contains diphosphatidylglycerol, phosphatidylethanolamine, phosphatidylglycerol, phosphatidylinositol, phosphatidylmethylethanolamine and phosphatidylinositol mannoside. The major fatty acids are C_18:1_*ω*9c, iso-C_16:0_, C_16:0_, iso-C_15:0_, and C_16:1_*ω*7c. The basic whole-cell sugar pattern includes arabinose, glucose and ribose. The genomic G + C content is 74%–75%. The type species of *Goekera* is *Goekera deserti*, sp. nov.

### Description of *Goekera deserti* comb. nov.

*G*. de.ser’ti (L. gen. Neut. n. *deserti*, of a desert, where the organisms were acquired).

Basonym: *Modestobacter deserti* Jiang et al. (2021).

The properties are as given in the species description by Jiang et al. (2021) with the following modification. According to genomic data, anaerobiosis and acetogenesis may occur. A repertoire of genes related to flagellum synthesis, chemotaxis, spore production and pilus assembly were annotated. Four different autotrophic mechanisms including the Wood-Ljungdahl pathway, C4-dicarboxylic acid and reductive citric acid cycles and carbonic anhydrases as well as a range of genes involved in the degradation of complex carbohydrates were also identified.

The accession number for the whole genome sequence of strain CPCC 205119^T^ is JAAGWK000000000.

The type strain CPCC 205119^T^ = I12A-02624^T^ = KCTC 49201^T^ = NBRC 113528^T^ was isolated from moss-dominated soil crusts collected from Shapotou NDER in Tengger Desert, China.

### Emended description of *Blastococcus aggregatus*

The properties are as given in the species description by Ahrens and Moll (1970) and emendations by Urzì et al. (2004) and Hezbri et al. (2016) with the following emendation. The genomic G + C content is 73.3%. The genome size is 4.6 Mbp. According to genomic data, anaerobiosis and acetogenesis may occur. A repertoire of genes related to flagellum synthesis, chemotaxis, spore production and pilus assembly were annotated. Three different autotrophic mechanisms including the Wood-Ljungdahl pathway, reductive citric acid cycle, and carbonic anhydrases as well as a range of genes involved in the degradation of complex carbohydrates were also identified.

The accession number for the whole genome sequence of the type strain DSM 4725^T^ is OBQI00000000.

The type strain B15^T^ = ATCC 25902^T^ = DSM 4725^T^ = JCM 12602^T^ = NCIMB 1849^T^ was isolated from Baltic Sea.

### Emended description of *Blastococcus jejuensis*

The properties are as given in the species description by Lee (2006) and emendation by Hezbri et al. (2016) with the following emendation. The presence of phosphatidylmethylethanolamine listed by Lee (2006) and confirmed by Hezbri et al. (2016) was incorrectly annotated by the latter and it should be recognised as hydroxiphosphatidylethanolamine accordingly to its chromatographic mobility.

The type strain KST3-10^T^ = DSM 19597^T^ = JCM 15614^T^ = KCCM 42251^T^ = NRRL B-24440^T^ was isolated from sand sediment of a beach in Jeju, Korea.

### Emended description of *Blastococcus saxobsidens*

The properties are as given in the species description by Urzì et al. (2004) and emendation by Hezbri et al. (2016) with the following emendation. The genomic G + C content is 73.5%. The genome size is 4.5 Mbp. According to genomic data, anaerobiosis and acetogenesis may occur. A repertoire of genes related to flagellum synthesis, chemotaxis, spore production and pilus assembly were annotated. Four different autotrophic mechanisms including the Wood-Ljungdahl pathway, C4-dicarboxylic acid and reductive citric acid cycles and carbonic anhydrases as well as a range of genes involved in the degradation of complex carbohydrates were also identified.

The accession number for the whole genome sequence of the type strain DSM 44509^T^ is SHKV01000001.

The type strain BC444^T^ = DSM 44509^T^ = JCM 13239^T^ = NRRL B-24246^T^ was isolated from from the surface of marble and calcareous stones in Italy.

### Emended description of *Blastococcus xanthilyniticus*

The properties are as given in the species description by Hezbri et al. (2018) with the following emendation. The genomic G + C content is 74.4%. The genome size is 4.6 Mbp. According to genomic data, anaerobiosis and acetogenesis may occur. A repertoire of genes related to flagellum synthesis, chemotaxis, spore production and pilus assembly were annotated. Four different autotrophic mechanisms including the Wood-Ljungdahl pathway, C4-dicarboxylic acid and reductive citric acid cycles and carbonic anhydrases as well as a range of genes involved in the degradation of complex carbohydrates were also identified.

The accession number for the whole genome sequence of the type strain DSM 46842^T^ is VNHW00000000.

The type strain BMG 862^T^ = DSM 46842^T^ = CECT 8884^T^ was isolated from a marble sample collected from the Bulla Regia monument, Northern Tunisia.

### Description of *Blastococcus aurantiacus* sp. nov.

*Blastococcus aurantiacus* (au.ran.ti.a’cus. N.L. masc. Adj. *aurantiacus* orange-coloured, referring to the orange colour of the colonies).

Colonies are bright red orange-coloured, opaque with a dry surface and regular margin. Cells are Gram-reaction-positive and catalase and oxidase negative cocci (0.6–1.6 μm in diameter) with a tendency to form aggregates. It grows in aerobiosis but, according to genomic data, anaerobiosis and acetogenesis may occur. Reproduction by budding is predominant but binary fission is also observed. Cells are non-motile and non-spore-forming but a repertoire of genes related to flagellum synthesis, chemotaxis, spore production and pilus assembly were annotated. Degradation for casein, tyrosine, starch, xanthine, and hypoxanthine are negative. Temperature and pH ranges are 15–37°C (optimal range) and 6.0–12.0 (optimum 6.5–8.0), respectively. NaCl is not needed for growth. It can grow between 0% and 4% NaCl (w/v; optimal range) but not at 8%. It grows well on GYM *Streptomyces*, R2A (DSMZ Medium 830), tryticase soy agar (TSA), Luedemann (DMSZ medium 877), and PYGV (DSMZ medium 621) media. According to API ZYM strips, the following enzymatic activities are present: esterase lipase (C8), lipase (C14), leucine arylamidase, valine acrylamidase, cysteine acrylamidase, trypsin, and α-galatosidase. According to the Biolog System, it oxidises: dextrin, D-maltose, D-trehalose, D-cellobiose, *β*-gentiobiose, sucrose, stachyose, *α*-D-lactose, *β*-methyl-D-glucoside, D-salicin, N-acetyl-D-glucosamine, N-acetyl-*β*-D-mannosamine, N-acetyl-D-galactosamine, 3-*O*-methyl-D-glucose, D-fucose, L-fucose, glycerol, D-glucose-6-phosphate, rifamycin SV, D-galacturonic acid, D-gluconic acid, D-glucuronic acid, D-saccharic acid, *α*-keto-glutaric acid, D-malic acid, L-malic acid, nalidixic acid, lithium chloride, potassium tellurite, tween 40, *γ*-amino-n-butyric acid, acetoacetic acid, propionic acid, acetic acid, aztreonam, butyric acid, and sodium bromate but not D-mannose, D-galactose, L-rhamnose, sodium lactate, fusidic acid, D-mannitol, D-arabitol, D-aspartic acid, D-serine, troleandomycin, minocycline, glycine-proline, L-alanine, L-arginine, L-glutamic acid, L-histidine, L-pyroglutamic acid, L-serine, lincomycin, guanidine hydrochloride, niaproof, pectin, L-galactonic acid-*γ*-lactone, glucuronamide, mucic acid, quinic acid, vancomycin, tetrazolium violet, tetrazolium blue, *p*-hydroxy-phenylacetic acid, methyl pyruvate, L-lactic acid, citric acid, bromo-succinic acid, *β*-hydroxy-butyric acid, *α*-keto-butyric acid, and sodium formate. In correlation, a range of genes involved in the degradation of complex carbohydrates were identified (chemoheterotrophy). Three different autotrophic mechanisms including the Wood-Ljungdahl pathway, reductive citric acid cycle and carbonic anhydrases were besides annotated (chemolithoautotrophy). Predominant fatty acids are C_18:1_*ω*8c, iso-C_16:0_, iso-C_16:1_ H, iso-C_15:0_, and iso-C_16:1_ H. The cell wall peptidoglycan contains *meso*-diaminopimelic acid. Galactose, glucose, and arabinose are the whole-cell sugars. Polar lipid profile consists of diphosphatidylglycerol, phosphatidylglycerol, phosphatidylethanolamine, phosphatidylcholine and phosphatidylinositol, glycophosphatidylinositol, two unidentified phospholipids, and three unidentified gylcolipids. The high-quality draught genome of strain DSM 44268^T^ was resolved to 18 scaffolds consisting of 4,163,046 bp, with a G + C content of 73.6%, 4,044 candidate protein-coding genes, 47 tRNA genes, and eight rRNA regions.

The INSDC accession number for the 16S rRNA gene sequences of the type strain AT 7-1^T^ (=DSM 44268^T^ = JCM 18931^T^) isolated from soil in the Atacama Desert (Chile) is MH479060. The IMG accession number for the whole genome sequence of strain DSM 44268^T^ is 2599185358.

### Description of *Blastococcus fimeti* sp. nov.

*Blastococcus fimeti* (fi.me’ti. L. neut. Gen. n. *fimeti*, of or from a dunghill).

Colonies are pink red-coloured, opaque with a greasy surface and irregular margin. Cells are Gram-reaction-positive and catalase and oxidase negative cocci (0.6–1.6 μm in diameter) with a tendency to form aggregates. It grows in aerobiosis but, according to genomic data, anaerobiosis and acetogenesis may occur. Reproduction by budding is predominant but binary fission is also observed. Cells are non-motile and non-spore-forming but a repertoire of genes related to flagellum synthesis, chemotaxis, spore production and pilus assembly were annotated. Degradation for casein, tyrosine, starch, xanthine, and hypoxanthine are negative and aesculin positive. Temperature and pH ranges are 15°C–37°C (optimal range) and 6.0–12.0 (optimum 7.0–9.0), respectively. NaCl is not needed for growth. It can grow between 0–4% NaCl (w/v; optimal range) but not at 8%. It grows well on GYM *Streptomyces*, R2A (DSMZ Medium 830), tryticase soy agar (TSA), Luedemann (DMSZ medium 877), GPHF (DSMZ medium 553), and PYGV (DSMZ medium 621) media. According to API ZYM strips, the following enzymatic activities are present: esterase lipase (C8), lipase (C14), leucine arylamidase, valine arylamidase, *α-*fucosidase. According to the Biolog System, it oxidises: dextrin, D-maltose, D-trehalose, D-cellobiose, sucrose, turanose, *β*-methyl-D-glucoside, D-salicin, N-acetyl-D-glucosamine, N-acetyl-*β*-D-mannosamine, D-glucose, 3-*O*-methyl-D-glucose, L-fucose, glycerol, D-glucose-6-phosphate, rifamycin SV, L-glutamic acid, methyl pyruvate, D-lactic acid methyl ester, citric acid, *α*-keto-glutaric acid, nalidixic acid, lithium chloride, potassium tellurite, tween 40, γ-amino-*n*-butyric acid, *β-*hydroxy-butyric acid, *α*-keto-butyric acid, acetoacetic acid, propionic acid, acetic acid, aztreonam, butyric acid, and sodium bromate but not D-raffinose, *α*-D-lactose, N-acetyl-D-galactosamine, N-acetyl-neuraminic acid, D-mannose, L-rhamnose, fusidic acid, D-serine (inhibitory concentration), D-arabitol, myo-inositol, D-fructose-6-phosphate, D-aspartic acid, D-serine, troleandomycin, minocycline, glycine-proline, L-alanine, L-arginine, L-histidine, L-pyroglutamic acid, L-serine, lincomycin, guanidine, hydrochloride, niaproof, L-galactonic acid-*γ*-lactone, mucic acid, quinic acid, D-saccharic acid, vancomycin, tetrazolium violet, tetrazolium blue, p-hydroxy-phenylacetic acid, L-lactic acid, L-malic, acid, bromo-succinic acid and sodium formate. In correlation, a range of genes involved in the degradation of complex carbohydrates were identified (chemoheterotrophy). Three different autotrophic mechanisms including the Wood-Ljungdahl pathway, reductive citric acid cycle and carbonic anhydrases were besides annotated (chemolithoautotrophy). Predominant fatty acids are iso-C_16:0_, anteiso-C_17:0_, anteiso-C_17:1_ C, iso-C_16:1_ H and C_18:1_*ω*9c. The cell wall peptidoglycan contains *meso*-diaminopimelic acid. Ribose, xylose, arabinose, mannose, and glucose are the whole-cell sugars. Polar lipid profile consists of diphosphatidylglycerol, phosphatidylethanolamine, phosphatidylcholine and phosphatidylinositol. The high-quality draught genome of strain DSM 44205^T^ was resolved to 30 scaffolds consisting of 4,044,261 bp, with a G + C content of 73.4%, 3,853 candidate protein-coding genes, 70 tRNA genes, and nine rRNA regions.

The INSDC accession number for the 16S rRNA gene sequences of the type strain G1S^T^ (=DSM 44205^T^ = CECT 8406^T^) isolated from seepage water of a dumping ground in Vancouver (Canada) is MH479059. The IMG accession number for the whole genome sequences of strain DSM 44205^T^ is 2599185193.

### Description of *Blastococcus haudaquaticus* sp. nov.

*Blastococcus haudaquaticus* (hau.da.qua’ti.cus. L. adv. *Haud*, not at all, by no means; L. masc. Adj. *aquaticus*, living, growing, or found in or by the water, aquatic; N.L. masc. Adj. *haudaquaticus*, growing far away from or without any water).

Colonies are reddish-brown coloured, opaque with a mucoid surface and regular margin. Cells are aerobic, Gram-reaction-positive and catalase and oxidase negative cocci (0.6–1.6 μm in diameter) with a tendency to form aggregates. It grows in aerobiosis but, according to genomic data, anaerobiosis and acetogenesis may occur. Reproduction by budding is predominant but binary fission is also observed. Cells are non-motile and non-spore-forming but a repertoire of genes related to flagellum synthesis, chemotaxis, spore production and pilus assembly were annotated. Degradation for casein, tyrosine, starch, xanthine, and hypoxanthine are negative. Temperature and pH ranges are 15–37°C (optimal range) and pH 5.0–11.0 (optimum 6.5–8.0), respectively. NaCl is not needed for growth. It can grow between 0% and 8% NaCl (w/v; optimal range). It grows well on GYM *Streptomyces*, R2A (DSMZ Medium 830), tryticase soy agar (TSA), Luedemann (DMSZ medium 877), and PYGV (DSMZ medium 621) media. According to API ZYM strips, the following enzymatic activities are present: esterase lipase (C8), lipase (C14), leucine arylamidase, valine acrylamidase, trypsin, and cystine acrylamidase. According to the Biolog System, it oxidises: dextrin, D-maltose, D-trehalose, D-cellobiose, *β*-gentiobiose, sucrose, turanose, *β*-methyl-D-glucoside, D-salicin, N-acetyl-D-glucosamine, D-glucose, D-fructose, sodium lactate, D-sorbitol, glycerol, rifamycin SV, L-aspartic acid, L-glutamic acid, L-histidine, mucic acid, L-lactic acid, citric acid, L-malic acid, lithium chloride, potassium tellurite, *γ*-amino-*n*-butyric acid, propionic acid, acetic acid, sodium formate, aztreonam, butyric acid, and sodium bromate but not *α*-D-lactose, N-acetyl-D-galactosamine, N-acetyl-neuraminic acid, 3-*O*-methyl-D-glucose, D-fucose, L-fucose, L-rhamnose, inosine, fusidic acid, D-serine (inhibitory concentration), D-arabitol, myo-inositol, D-aspartic acid, D-serine, troleandomycin, minocycline, glycine-proline, L-arginine, L-pyroglutamic acid, lincomycin, guanidine hydrochloride, niaproof, L-galactonic acid-*γ*-lactone, D-glucuronic acid, glucuronamide, quinic acid, vancomycin, tetrazolium violet, tetrazolium blue, p-hydroxy-phenylacetic acid, D-lactic acid methyl ester, *α*-keto-glutaric acid, D-malic acid, bromo-succinic acid, nalidixic acid, *α*-keto-butyric acid, and acetoacetic acid. In correlation, a range of genes involved in the degradation of complex carbohydrates were identified (chemoheterotrophy). Three different autotrophic mechanisms including the Wood-Ljungdahl pathway, reductive citric acid cycle and carbonic anhydrases were besides annotated (chemolithoautotrophy). Predominant fatty acids are iso-C_15:0_, iso-C_16:1_ H, iso-C_16:0_, C_17:1_*ω*8c, C_18:1_*ω*9c, and C_16:1_*ω*7c. The cell wall peptidoglycan contains *meso*-diaminopimelic acid. Ribose, mannose, galactose, and glucose are the whole-cell sugars. Polar lipids consist of diphosphatidylglycerol, phosphatidylethanolamine, phosphatidylglycerol, phosphatidylcholine, and phosphatidylinositol. The high-quality draught genome of strain DSM 44270^T^ was resolved to 11 scaffolds consisting of 4,512,672 bp, with a G + C content of 73.29%, 4,410 candidate protein-coding genes, 48 tRNA genes, and six rRNA regions.

The INSDC accession number for the 16S rRNA gene sequences of the type strain AT 7-14^T^ (=DSM 44270^T^ = JCM 18932^T^) isolated from soil in the Atacama Desert (Chile) is MH479062. The IMG accession number for the whole genome sequences of strain DSM 44270^T^ is 2728369258.

### Description of *Blastococcus mobilis* sp. nov.

*Blastococcus mobilis* (mo’bi.lis. L. masc. Adj. *mobilis* movable, motile).

Colonies are reddish-brown coloured, opaque with a mucoid surface and regular margin. Cells are aerobic, Gram-reaction-positive and catalase and oxidase negative cocci (0.6–1.6 μm in diameter) with a tendency to form aggregates. It grows in aerobiosis but, according to genomic data, anaerobiosis and acetogenesis may occur. Reproduction by budding is predominant but binary fission is also observed. Cells are non-motile and non-spore-forming but a repertoire of genes related to flagellum synthesis, chemotaxis, spore production and pilus assembly were annotated. Degradation for casein, tyrosine, starch, xanthine, and hypoxanthine are negative. Temperature and pH ranges are 15–37°C (optimal range) and pH 6.5–11.0 (optimum 6.0–10.0), respectively. NaCl is not needed for growth. It can grow between 0% and 1% (w/v; optimal range) but not at 4%. It grows well on GYM *Streptomyces*, R2A (DSMZ Medium 830), Luedemann (DMSZ medium 877), and PYGV (DSMZ medium 621) media. According to API ZYM strips, the following enzymatic activities are present: esterase (C4), esterase lipase (C8), lipase (C14), leucine arylamidase, and valine arylamidase. According to the Biolog System, it oxidises: dextrin, D-maltose, sucrose, turanose, *β*-methyl-D-glucoside, D-glucose, D-mannose, D-fructose, D-galactose, sodium lactate, D-sorbitol, D-mannitol, D-arabitol, myo-inositol, glycerol, L-pyroglutamic acid, pectin, D-glucuronic acid, quinic acid, methyl pyruvate, D-lactic acid methyl ester, L-lactic acid, nalidixic acid, potassium tellurite, tween 40, *α*-hydroxy-butyric acid, *β*-hydroxy-butyric acid, *β*-keto-butyric acid, acetoacetic acid, propionic acid, acetic acid, aztreonam, butyric acid, and sodium bromated but not D-raffinose, *α*-D-lactose, D-melibiose, N-acetyl-D-galactosamine, N-acetyl-neuraminic acid, L-fucose, L-rhamnose, inosine, fusidic acid, D-serine (inhibitory concentration), D-aspartic acid, D-serine, troleandomycin, rifamycin SV, minocycline, gelatin, glycine-proline, L-histidine, L-serine, lincomycin, guanidine hydrochloride, niaproof, L-galactonic acid-*γ*-lactone, mucic acid, D-saccharic acid, vancomycin, tetrazolium violet, tetrazolium blue, p-hydroxy-phenylacetic acid, citric acid, D-malic acid, L-malic acid, bromo-succinic acid, *γ*-amino-*n*-butyric acid, and sodium formate. In correlation, a range of genes involved in the degradation of complex carbohydrates were identified (chemoheterotrophy). Three different autotrophic mechanisms including the Wood-Ljungdahl pathway, reductive citric acid cycle and carbonic anhydrases were besides annotated (chemolithoautotrophy). Predominant fatty acids are iso-C_15:0_, iso-C_16:1_ H, iso-C_16:0_, C_17:1_ *ω*8c, C_18:1_ *ω*9c, and C_16:1_ *ω*7c. The cell wall peptidoglycan contains *meso*-diaminopimelic acid. Rhamnose, ribose, mannose, arabinose, galactose, and glucose are the whole-cell sugars. Polar lipid profile consists of diphosphatidylglycerol, phosphatidylethanolamine, phosphatidylglycerol, phosphatidylcholine, and phosphatidylinositol. The high-quality draught genome of strain DSM 44272^T^ was resolved to 88 scaffolds consisting of 5,094,633 bp, with a G + C content of 72.5%, 4,938 candidate protein-coding genes, 49 tRNA genes, and six rRNA regions.

The INSDC accession number for the 16S rRNA gene sequences of the type strain AT 7(−2)-11^T^ (=DSM 44272^T^ = JCM 18933^T^) isolated from soil in the Atacama Desert (Chile) is MH479063. The IMG accession number for the whole genome sequences of strain DSM 44272^T^ is 2724679778.

## Data availability statement

The datasets presented in this study can be found in online repositories. The names of the repository/repositories and accession number(s) can be found in the article/Supplementary material.

## Author contributions

MdCM-C designed the study. MdCM-C, AY, MR, and PS performed experiments. MdCM-C, AY, and JM-K performed bioinformatics analyses and wrote the manuscript. H-PK funded the study. All authors contributed to the article and approved the submitted version.

## Funding

MdCM-C was the recipient of a DSMZ postdoctoral fellowship 2013-2015. MdCM-C is grateful for funding received from the Ramón y Cajal Research Grant (RYC2019-028468-I) from the Spanish Ministry of Economy, Industry and Competitiveness (MINECO). The work conducted by the Joint Genome Institute, a U.S. Department of Energy Office of Science User Facility, is supported under contract no. DE-AC02-05CH11231.

## Conflict of interest

The authors declare that the research was conducted in the absence of any commercial or financial relationships that could be construed as a potential conflict of interest.

## Publisher’s note

All claims expressed in this article are solely those of the authors and do not necessarily represent those of their affiliated organizations, or those of the publisher, the editors and the reviewers. Any product that may be evaluated in this article, or claim that may be made by its manufacturer, is not guaranteed or endorsed by the publisher.
