# Corrigendum: Genotype–phenotype correlations within the *Geodermatophilaceae*

**DOI:** 10.3389/fmicb.2022.1100319

**Published:** 2023-01-20

**Authors:** Maria del Carmen Montero-Calasanz, Adnan Yaramis, Manfred Rohde, Peter Schumann, Hans-Peter Klenk, Jan P. Meier-Kolthoff

**Affiliations:** ^1^IFAPA Las Torres-Andalusian Institute of Agricultural and Fisheries Research and Training, Junta de Andalucía, Seville, Spain; ^2^School of Natural and Environmental Sciences, Newcastle University, Newcastle upon Tyne, United Kingdom; ^3^Central Facility for Microscopy, HZI – Helmholtz Centre for Infection Research, Braunschweig, Germany; ^4^Leibniz Institute DSMZ – German Collection of Microorganisms and Cell Cultures, Braunschweig, Germany; ^5^Department Bioinformatics and Databases, Leibniz Institute DSMZ – German Collection of Microorganisms and Cell Cultures, Braunschweig, Germany

**Keywords:** *Trujillonella*, *Pleomorpha*, *Goekera*, phylogenetic systematics, *in silico* chemotaxonomy

In the published article, there were some nomenclatural errors in the protologues of *Trujillonella* and *Trujillonella endophytica, Pleomorpha* and *Pleomorpha daqingensis, Goekera* and *Goekera deserti* and *Blastococcus xantinilythicus*.

A correction has been made to the section **Final remarks and taxonomic consequences**, as follows:

**Description of** ***Trujillonella*** **gen. nov**.

Tru.jil.lo.nel'la. N.L. fem. dim. n. *Trujillonella* named in honour of Martha E. Trujillo in recognition of her contributions to microbial systematics, mainly on Actinobacteria, on Bergey's Manual trust, and as the Editor-in-chief of the International Journal of Systematic and Evolutionary Microbiology.

Cells are aerobic, non-motile, non-spore-forming, Gram-stain positive, catalase-positive and oxidase-negative. Cells occur singly, in pairs or in tetrads, often tending to form aggregates. The peptidoglycan in the cell-wall contains *meso*-diaminopimelic acid. The predominant menaquinone is MK-9(H_4_), with MK-8 and MK-9(H_6_) as minor components. The basic polar lipid profile contains diphosphatidylglycerol, phosphatidylcholine, phosphatidylethanolamine, and phosphatidylinositol. The major fatty acids are iso-C_16:0_, iso-C_15:0_ and C_18:1_ω9c. The basic whole-cell sugar pattern includes arabinose and galactose. The genomic G + C content is 71–72%. The type species of *Trujillonella* is *Trujillonella endophytica* comb. nov.

**Description of** ***Trujillonella endophytica*** **comb. nov**.

*T*. en.do.phy'ti.ca (Gr. pref. *endo-*, within; Gr. neut. n. *phyton*, plant; L. fem. adj. suff. –*ica*, adjectival suffix used with the sense of belonging to; N.L. fem. adj. *endophytica*, within plant, endophytic, pertaining to the isolation from plant tissues).

Basonym: *Blastococcus endophyticus* Zhu et al. 2013 emend. Hezbri et al. 2016.

The properties are as given in the species description by Zhu et al. (2013) and emendation by Hezbri et al. (2016) with the following modification. The genomic G + C content is 74.6%. The genome size is 4.9 Mbp. According to genomic data, anaerobiosis and acetogenesis may occur. A repertoire of genes related to flagellum synthesis, chemotaxis, spore production and pilus assembly were annotated. Four different autotrophic mechanisms including the Wood-Ljungdahl pathway, C4-dicarboxylic acid and reductive citric acid cycles and carbonic anhydrases as well as a range of genes involved in the degradation of complex carbohydrates were also identified.

The accession number for the whole genome sequence of strain DSM 45413^T^ is FOEE00000000.

The type strain YIM 68236^T^ = CCTCC AA 209045^T^ = DSM 45413^T^ = KCTC 19998^T^ was isolated from healthy leaves of *Camptotheca acuminata* collected in Yunnan Province, south-west China.

**Description of** ***Pleomorpha*** **gen. nov**.

Ple.o.mor'pha. Gr. adv. *pleon* more; Gr. fem. n. *morphe*, shape or form; N.L. fem. n. *Pleomorpha*, organism showing multiple forms.

Pleiomorphic, motile, spore-forming, aerobic, Gram-stain positive cells. Those occurs singly or associated in aggregates. The peptidoglycan in the cellwall contains *meso*-diaminopimelic acid. The predominant menaquinone is MK-9(H_4_). The basic polar lipid profile contains diphosphatidylglycerol, phosphatidylcholine, phosphatidylglycerol, phosphatidylethanolamine, and phosphatidylinositol. The major fatty acids are iso-C_16:0_ and iso-C_15:0_. The basic whole-cell sugar pattern includes galactose, glucose and xylose. The genomic G + C content is 73–74%. The type species of *Pleomorpha* is *Pleomorpha daqingensis* comb. nov.

**Description of** ***Pleomorpha daqingensis*** **comb. nov**.

*P*. da.qing.en'sis (N.L. fem. adj. *daqingensis*, pertaining to Daqing city, China, where the type strain was isolated).

Basonym: *Geodermatophilus daqingensis* Wang et al. 2017.

The properties are as given in the species description by Wang et al. (2017) with the following modification. The genomic G + C content is 73.6%. The genome size is 5.4 Mbp. According to genomic data, anaerobiosis and acetogenesis may occur. A repertoire of genes related to flagellum synthesis, chemotaxis, spore production and pilus assembly were annotated. Four different autotrophic mechanisms including the Wood-Ljungdahl pathway, C4-dicarboxylic acid and reductive citric acid cycles and carbonic anhydrases as well as a range of genes involved in the degradation of complex carbohydrates were also identified.

The accession number for the whole genome sequence of strain DSM 104001^T^ is JACBZT000000000.

The type strain WT-2-1^T^ = CGMCC 4.7381^T^ = DSM 104001^T^ was isolated from petroleum- contaminated soil in Daqing city, China.

**Description of** ***Goekera*** **gen. nov**.

Goe'ke.ra. N.L. fem. n. *Goekera*, named in honour of Markus Göker in recognition of his contributions to microbial systematics, including work on *Actinobacteria*, on the List of Prokaryotic Names with Standing in Nomenclature (LPSN), and as a member of the Judicial Commission.

Cells are motile, non-spore-forming, aerobic, Gram-stain positive, catalase and oxidase positive cocci and/short rods. Bud-like structure was observed for some cells. The peptidoglycan in the cell-wall contains *meso*-diaminopimelic acid. The predominant menaquinone is MK-9(H_4_), with MK-8(H_4_) as a minor component. The basic polar lipid profile contains diphosphatidylglycerol, phosphatidylethanolamine, phosphatidylglycerol, phosphatidylinositol, phosphatidylmethylethanolamine and phosphatidylinositol mannoside. The major fatty acids are C_18:1_ω9c, iso-C_16:0_, C_16:0_, iso-C_15:0_, and C_16:1_ω7c.The basic whole-cell sugar pattern includes arabinose, glucose and ribose. The genomic G + C content is 74–75%. The type species of *Goekera* is *Goekera deserti* comb. nov.

**Description of** ***Goekera deserti*** **comb. nov**.

*G*. de.ser'ti (L. gen. neut. n. *deserti*, of a desert, where the organisms were acquired).

Basonym: *Modestobacter deserti* Jiang et al. 2023.

The properties are as given in the species description by Jiang et al. (2021) with the following modification. According to genomic data, anaerobiosis and acetogenesis may occur. A repertoire of genes related to flagellum synthesis, chemotaxis, spore production and pilus assembly were annotated. Four different autotrophic mechanisms including the Wood-Ljungdahl pathway, C4-dicarboxylic acid and reductive citric acid cycles and carbonic anhydrases as well as a range of genes involved in the degradation of complex carbohydrates were also identified.

The accession number for the whole genome sequence of strain CPCC 205119^T^ is JAAGWK000000000.

The type strain CPCC 205119^T^ = I12A-02624^T^ = KCTC 49201^T^ = NBRC 113528^T^ was isolated from moss-dominated soil crusts collected from Shapotou NDER in Tengger Desert, China.

**Emended description of** ***Blastococcus xanthinilyticus*** **Hezbri et al. (2018)**

The properties are as given in the species description by Hezbri et al. (2018) with the following emendation. The genomic G + C content is 74.4%. The genome size is 4.6 Mbp. According to genomic data, anaerobiosis and acetogenesis may occur. A repertoire of genes related to flagellum synthesis, chemotaxis, spore production and pilus assembly were annotated. Four different autotrophic mechanisms including the Wood-Ljungdahl pathway, C4-dicarboxylic acid and reductive citric acid cycles and carbonic anhydrases as well as a range of genes involved in the degradation of complex carbohydrates were also identified.

The accession number for the whole genome sequence of the type strain DSM 46842^T^ is VNHW00000000.

The type strain BMG 862^T^ = DSM 46842^T^ = CECT 8884^T^ was isolated from a marble sample collected from the Bulla Regia monument, Northern Tunisia.

The authors apologize for this error and state that this does not change the scientific conclusions of the article in any way.
