# Supplementary material for: Genotype–phenotype correlations within the Geodermatophilaceae
Source: Front Microbiol. 2022 Nov 10;13:975365. doi: 10.3389/fmicb.2022.975365 (PMC9686282; doi:10.3389/fmicb.2022.975365)
Supplement: Supplementary file 1 [file Presentation_1.pptx]

## Slide 1
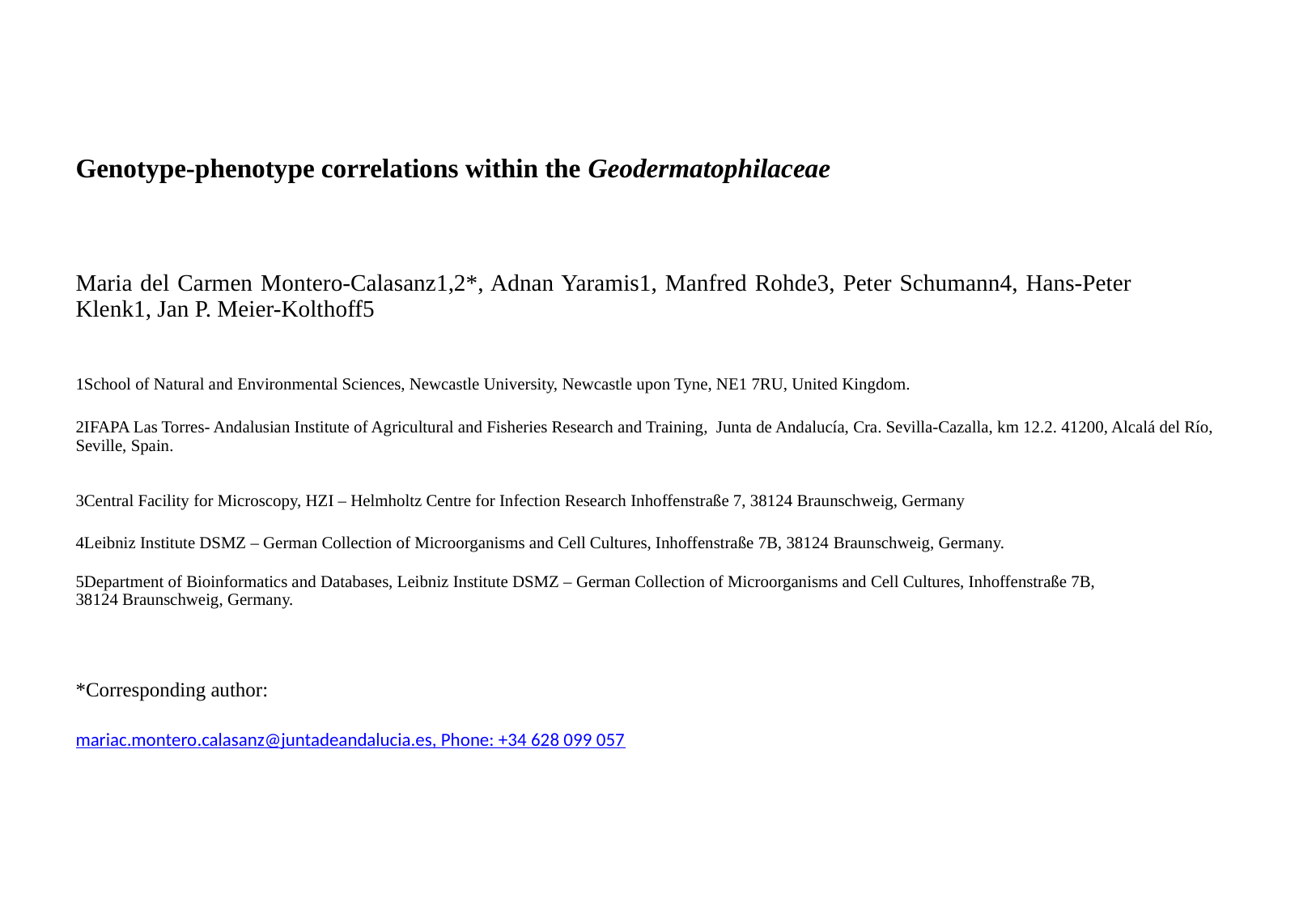

| Genotype-phenotype correlations within the Geodermatophilaceae | |
| --- | --- |
| | |
| Maria del Carmen Montero-Calasanz1,2\*, Adnan Yaramis1, Manfred Rohde3, Peter Schumann4, Hans-Peter Klenk1, Jan P. Meier-Kolthoff5 | |
| | |
| 1School of Natural and Environmental Sciences, Newcastle University, Newcastle upon Tyne, NE1 7RU, United Kingdom. | |
| 2IFAPA Las Torres- Andalusian Institute of Agricultural and Fisheries Research and Training, Junta de Andalucía, Cra. Sevilla-Cazalla, km 12.2. 41200, Alcalá del Río, Seville, Spain. | |
| 3Central Facility for Microscopy, HZI – Helmholtz Centre for Infection Research Inhoffenstraße 7, 38124 Braunschweig, Germany | |
| 4Leibniz Institute DSMZ – German Collection of Microorganisms and Cell Cultures, Inhoffenstraße 7B, 38124 Braunschweig, Germany. | |
| 5Department of Bioinformatics and Databases, Leibniz Institute DSMZ – German Collection of Microorganisms and Cell Cultures, Inhoffenstraße 7B, 38124 Braunschweig, Germany. | |
| | |
| \*Corresponding author: | |
| mariac.montero.calasanz@juntadeandalucia.es, Phone: +34 628 099 057 | |

## Slide 2
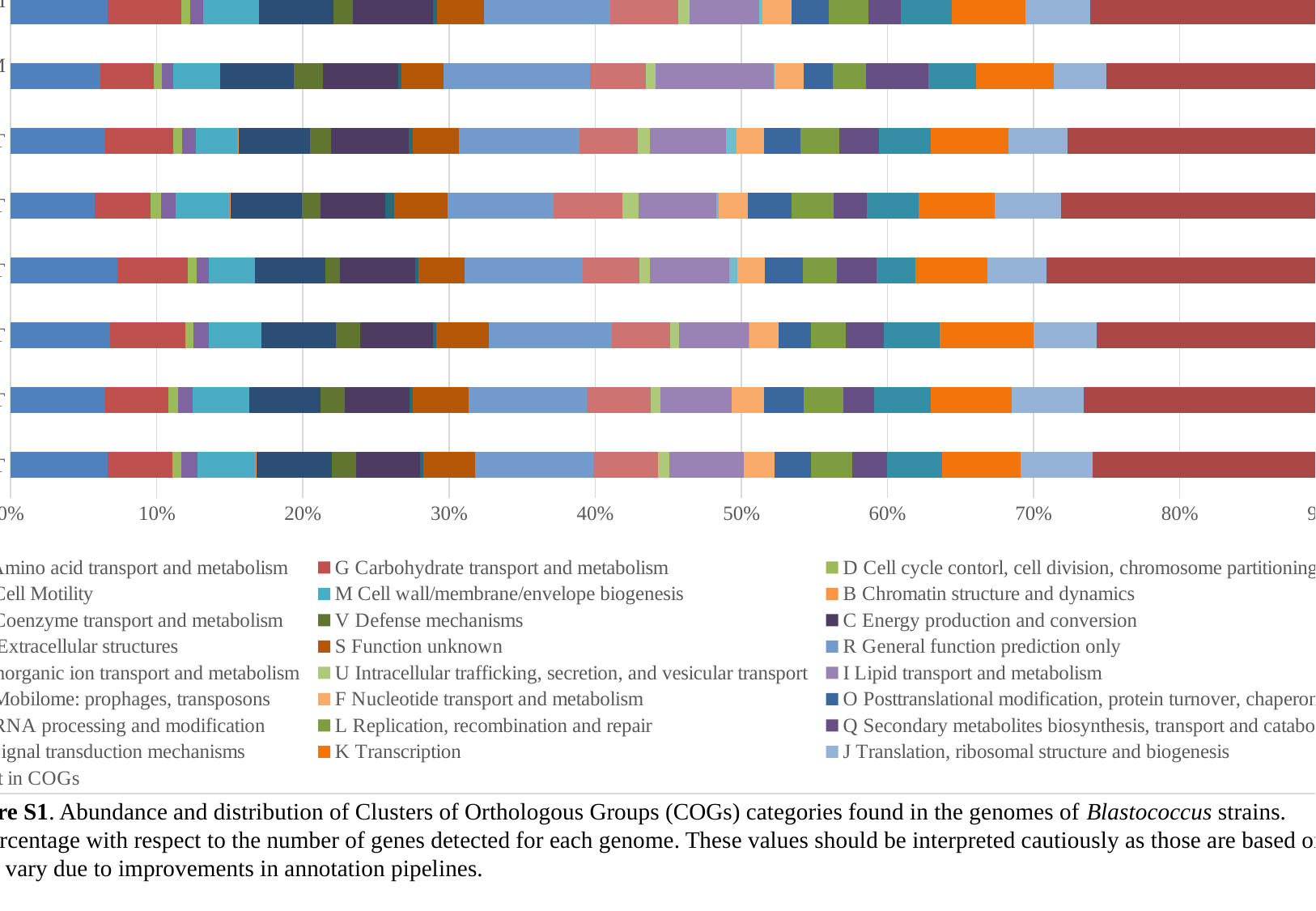

### Chart
| Category | E Amino acid transport and metabolism | G Carbohydrate transport and metabolism | D Cell cycle contorl, cell division, chromosome partitioning | N Cell Motility | M Cell wall/membrane/envelope biogenesis | B Chromatin structure and dynamics | H Coenzyme transport and metabolism | V Defense mechanisms | C Energy production and conversion | W Extracellular structures | S Function unknown | R General function prediction only | P Inorganic ion transport and metabolism | U Intracellular trafficking, secretion, and vesicular transport | I Lipid transport and metabolism | X Mobilome: prophages, transposons | F Nucleotide transport and metabolism | O Posttranslational modification, protein turnover, chaperones | A RNA processing and modification | L Replication, recombination and repair | Q Secondary metabolites biosynthesis, transport and catabolism | T Signal transduction mechanisms | K Transcription | J Translation, ribosomal structure and biogenesis | Not in COGs |
|---|---|---|---|---|---|---|---|---|---|---|---|---|---|---|---|---|---|---|---|---|---|---|---|---|---|
| B. sp. DSM 44205T | 9.0 | 5.970000000000001 | 0.81 | 1.46 | 5.4 | 0.07000000000000002 | 6.95 | 2.3099999999999996 | 5.84 | 0.3400000000000001 | 4.79 | 10.93 | 5.970000000000001 | 1.02 | 6.89 | 0.0 | 2.8499999999999996 | 3.3299999999999996 | 0.030000000000000006 | 3.84 | 3.19 | 5.09 | 7.23 | 6.6899999999999995 | 35.06 |
| B. sp. DSM 44268T | 8.83 | 5.89 | 0.8700000000000001 | 1.34 | 5.28 | 0.030000000000000006 | 6.619999999999999 | 2.24 | 6.09 | 0.30000000000000004 | 5.1499999999999995 | 11.07 | 5.89 | 0.9400000000000001 | 6.619999999999999 | 0.0 | 3.01 | 3.68 | 0.030000000000000006 | 3.71 | 2.88 | 5.28 | 7.49 | 6.76 | 36.13 |
| B. sp. DSM 44270T | 9.139999999999999 | 6.95 | 0.78 | 1.35 | 4.85 | 0.030000000000000006 | 6.83 | 2.25 | 6.68 | 0.33000000000000007 | 4.819999999999999 | 11.360000000000001 | 5.33 | 0.8700000000000001 | 6.41 | 0.06000000000000001 | 2.7 | 2.94 | 0.030000000000000006 | 3.18 | 3.54 | 5.119999999999999 | 8.629999999999999 | 5.81 | 34.57 |
| B. sp. DSM 44272T | 10.32 | 6.7700000000000005 | 0.8600000000000001 | 1.1599999999999997 | 4.470000000000001 | 0.030000000000000006 | 6.74 | 1.46 | 7.22 | 0.36000000000000004 | 4.41 | 11.39 | 5.52 | 0.9500000000000001 | 7.72 | 0.7200000000000001 | 2.65 | 3.7 | 0.030000000000000006 | 3.25 | 3.82 | 3.73 | 6.95 | 5.76 | 41.02 |
| B. aggregatus DSM 4725T | 7.970000000000001 | 5.33 | 1.03 | 1.35 | 5.21 | 0.06000000000000001 | 6.75 | 1.7700000000000002 | 6.17 | 0.8400000000000001 | 5.109999999999999 | 10.120000000000001 | 6.49 | 1.57 | 7.39 | 0.26 | 2.73 | 4.18 | 0.030000000000000006 | 3.9499999999999997 | 3.18 | 4.92 | 7.23 | 6.359999999999999 | 39.07 |
| B. colisei DSM 46837T | 8.98 | 6.41 | 0.8300000000000001 | 1.28 | 4.02 | 0.030000000000000006 | 6.73 | 2.05 | 7.319999999999999 | 0.4 | 4.33 | 11.4 | 5.53 | 1.1700000000000002 | 7.21 | 0.9400000000000001 | 2.65 | 3.3899999999999997 | 0.030000000000000006 | 3.7 | 3.73 | 4.9 | 7.35 | 5.609999999999999 | 38.21 |
| B. endophyticus DSM 45413T | 8.219999999999999 | 4.88 | 0.7100000000000001 | 1.01 | 4.27 | 0.030000000000000006 | 6.71 | 2.68 | 6.87 | 0.30000000000000004 | 3.8299999999999996 | 13.34 | 5.1499999999999995 | 0.8800000000000001 | 10.739999999999998 | 0.11000000000000001 | 2.63 | 2.66 | 0.030000000000000006 | 3.01 | 5.7 | 4.35 | 7.07 | 4.819999999999999 | 33.349999999999994 |
| B. saxobsidens DSM 44509T | 8.960000000000003 | 6.859999999999999 | 0.81 | 1.22 | 5.17 | 0.030000000000000006 | 6.89 | 1.7500000000000002 | 7.42 | 0.38000000000000006 | 4.39 | 11.69 | 6.2700000000000005 | 1.03 | 6.45 | 0.3400000000000001 | 2.66 | 3.48 | 0.030000000000000006 | 3.67 | 2.98 | 4.73 | 6.83 | 5.95 | 35.39 |
| B. xanthinilyticus DSM 46842T | 9.43 | 7.35 | 0.78 | 1.36 | 4.68 | 0.030000000000000006 | 6.64 | 2.57 | 6.51 | 0.4 | 4.59 | 10.729999999999999 | 5.359999999999999 | 1.05 | 6.359999999999999 | 0.2800000000000001 | 2.64 | 3.22 | 0.030000000000000006 | 3.38 | 3.19 | 4.9 | 8.65 | 5.859999999999999 | 37.59 |Supplementary Figure S1. Abundance and distribution of Clusters of Orthologous Groups (COGs) categories found in the genomes of Blastococcus strains.
Values are given in percentage with respect to the number of genes detected for each genome. These values should be interpreted cautiously as those are based on draft genomes. Numbers may equally vary due to improvements in annotation pipelines.

## Slide 3
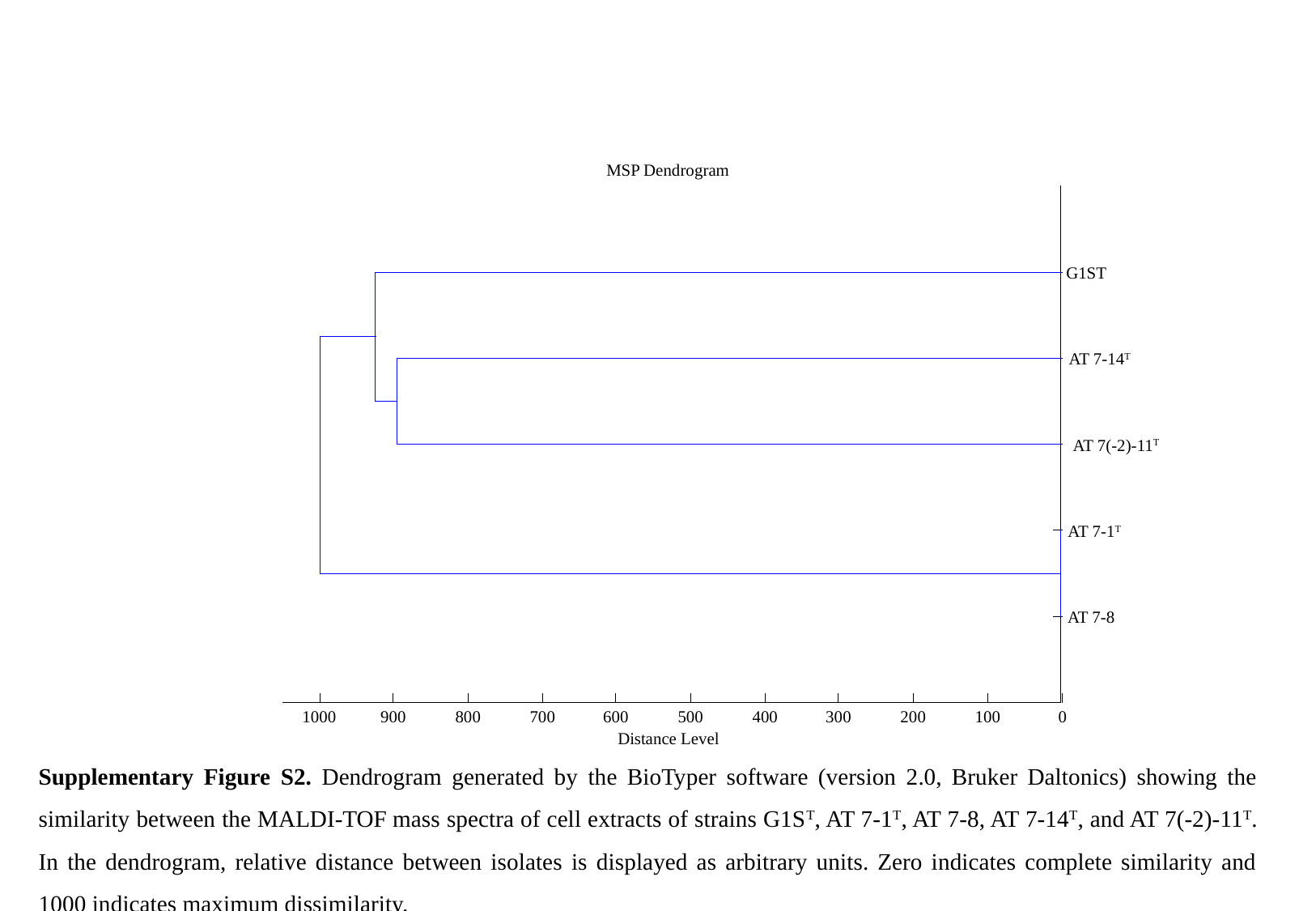

MSP Dendrogram
G1ST
AT 7-14T
 AT 7(-2)-11T
AT 7-1T
AT 7-8
1000
900
800
700
600
500
400
300
200
100
0
Distance Level
Supplementary Figure S2. Dendrogram generated by the BioTyper software (version 2.0, Bruker Daltonics) showing the similarity between the MALDI-TOF mass spectra of cell extracts of strains G1ST, AT 7-1T, AT 7-8, AT 7-14T, and AT 7(-2)-11T. In the dendrogram, relative distance between isolates is displayed as arbitrary units. Zero indicates complete similarity and 1000 indicates maximum dissimilarity.

## Slide 4
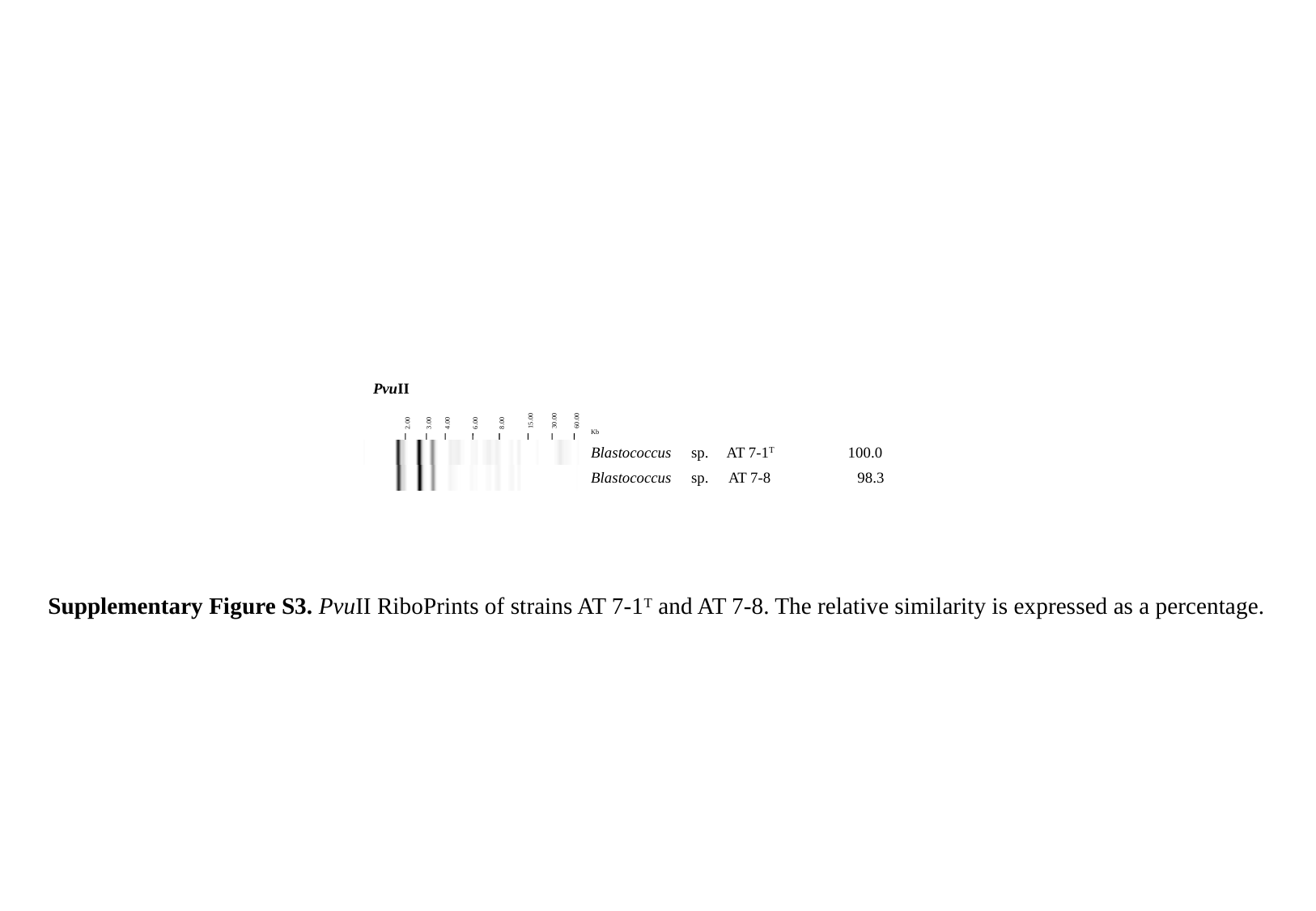

PvuII
15.00
30.00
60.00
3.00
4.00
6.00
8.00
2.00
Kb
Blastococcus
sp.
AT 7-1T	100.0
Blastococcus
sp.
AT 7-8	 98.3
Supplementary Figure S3. PvuII RiboPrints of strains AT 7-1T and AT 7-8. The relative similarity is expressed as a percentage.

## Slide 5
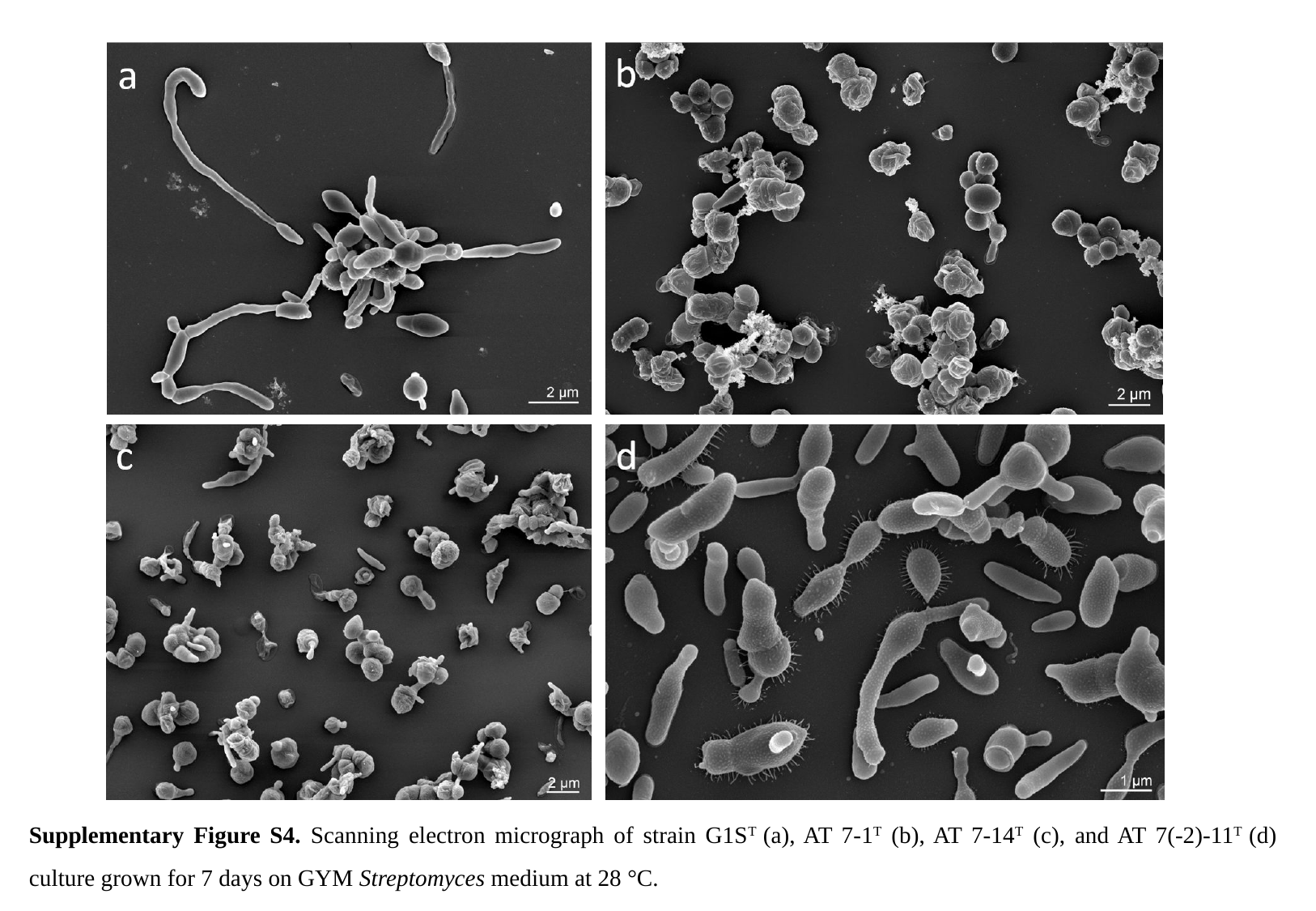

Supplementary Figure S4. Scanning electron micrograph of strain G1ST (a), AT 7-1T (b), AT 7-14T (c), and AT 7(-2)-11T (d) culture grown for 7 days on GYM Streptomyces medium at 28 °C.

## Slide 6
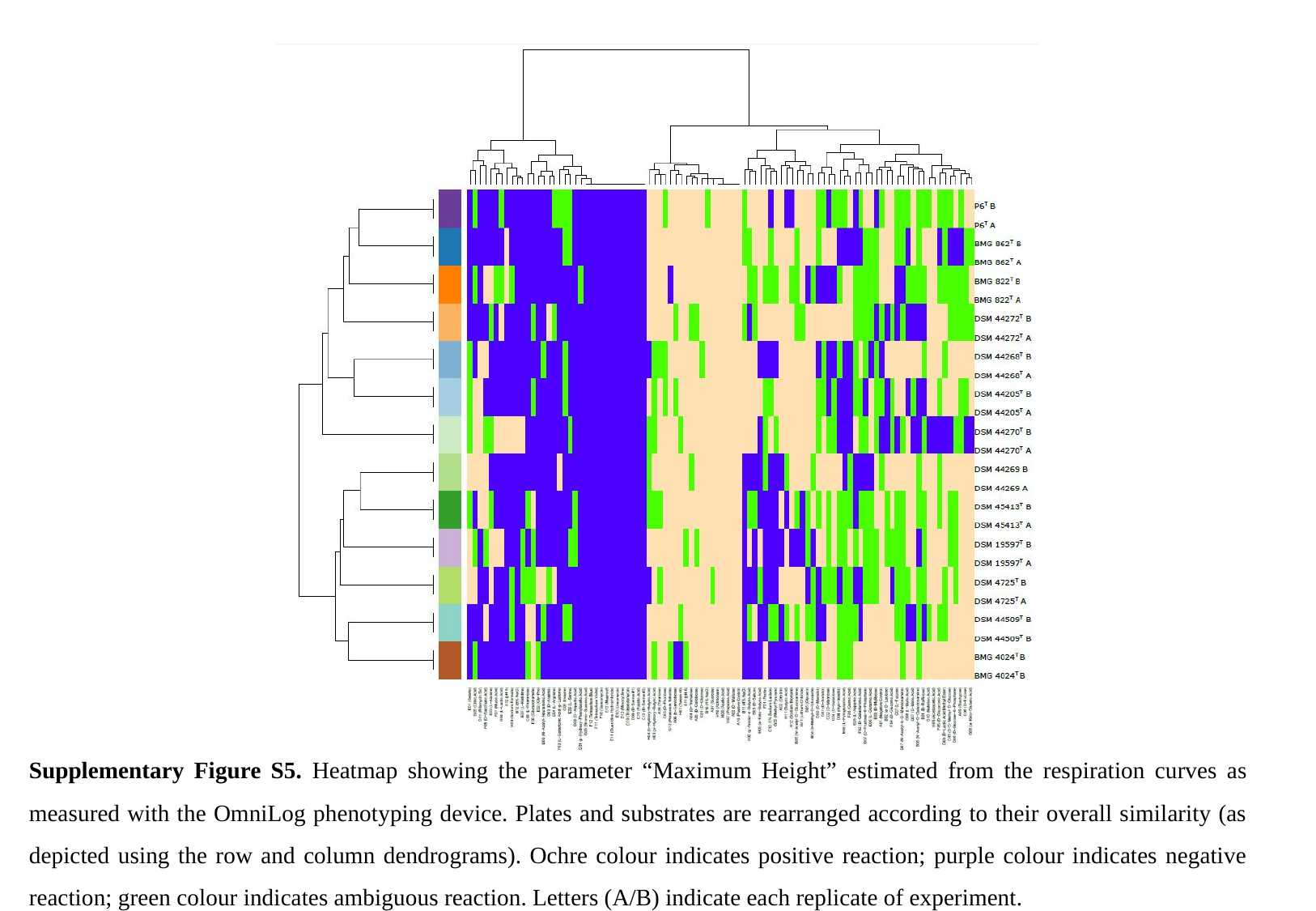

Supplementary Figure S5. Heatmap showing the parameter “Maximum Height” estimated from the respiration curves as measured with the OmniLog phenotyping device. Plates and substrates are rearranged according to their overall similarity (as depicted using the row and column dendrograms). Ochre colour indicates positive reaction; purple colour indicates negative reaction; green colour indicates ambiguous reaction. Letters (A/B) indicate each replicate of experiment.
